# Wnt-associated DKK3 in keratinocytes mediates radiation-induced hyperplasia, dermatitis and skin fibrosis

**DOI:** 10.1038/s41392-025-02541-z

**Published:** 2026-02-02

**Authors:** Li Li, Ramon Lopez Perez, Khuram Shehzad, Richard Jennemann, Claudia Schmidt, Thomas Walle, Alexandra Tietz-Dahlfuß, Elisabeth Grimm, Joscha A. Kraske, Peter Häring, Uladzimir Barayeu, Tobias P. Dick, Luxi Ye, Stephan A. Braun, Michael Hertl, Thomas Worzfeld, Thorsten Wiech, Huihui Ji, Jing Su, Jonathan M. Schneeweiss, Muzi Liu, Katharina Kommoss, Matthias Heikenwälder, Bingwen Zou, Sabrina Mücklich, Kerstin Steinbrink, Verena K. Raker, Wenjun Wu, Elfriede Noessner, Hermann-Josef Gröne, Peter J. Nelson, Roger Sandhoff, Peter E. Huber

**Affiliations:** 1 Molecular Radiooncology, German Cancer Research Center (DKFZ), Heidelberg, Germany; 2 Research Group Lipid Pathobiochemistry, German Cancer Research Center (DKFZ), Heidelberg, Germany; 3 Clinical Biochemistry, Internal Medicine IV, LMU University Hospital, Munich, Germany; 4 Dept. of Nephropathy, The Affiliated Hospital of Southwest Medical University and Sichuan Clinical Research Center for Nephropathology, Luzhou, China; 5 Light Microscopy Core Facility, German Cancer Research Center (DKFZ), Heidelberg, Germany; 6 Dept. of Medical Oncology, National Center for Tumor Diseases (NCT) & Heidelberg University Hospital, Medical Faculty Heidelberg, Heidelberg University, Heidelberg, Germany; 7 Faculty of Biosciences, Heidelberg University, Heidelberg, Germany; 8 Medical Physics in Radiation Oncology, German Cancer Research Center (DKFZ), Heidelberg, Germany; 9 Division of Redox Regulation, German Cancer Research Center (DKFZ), DKFZ-ZMBH Alliance, Heidelberg, Germany; 10 Dept. of Dermatology, University Hospital Münster, University of Münster, Münster, Germany; 11 Dept. of Dermatology, Medical Faculty, Heinrich-Heine University, Düsseldorf, Germany; 12 Dept. of Dermatology and Allergy, University Clinics Marburg, Marburg, Germany; 13 Institute of Pharmacology, Marburg University, Marburg, Germany; 14 Nephropathology University Clinics Hamburg-Eppendorf (UKE Hamburg), Hamburg, Germany; 15 Dept. of Pathology, School of Basic Medical Sciences, Peking University Third Hospital, Peking University Health Science Center, Beijing, China; 16 Chronic Inflammation and Cancer, German Cancer Research Center (DKFZ), Heidelberg, Germany; 17 Dept. of Dermatology, University Hospital Heidelberg, Heidelberg, Germany; 18 M3 Research Center for Malignome, Metabolome and Microbiome, Medical Faculty, University of Tuebingen, Tuebingen, Germany; 19 Dept. of Thoracic and Radiation Oncology and State Key Laboratory of Biotherapy, Cancer Center, West China Hospital; West China School of Basic Medical Sciences & Forensic Medicine, Sichuan University, Chengdu, China; 20 Cells in Motion Interfaculty Center and Department of Dermatology, University of Münster, Münster, Germany; 21 Dept. of Radiology, Union Hospital, Tongji Medical College, Huazhong University of Science and Technology, Wuhan, China; 22 Hubei Province Key Laboratory of Molecular Imaging, Wuhan, China; 23 Immunoanalytics-Tissue Control Immunocytes, Helmholtz Center, Munich, Germany; 24 Medical Faculty, University of Heidelberg, Heidelberg, Germany; 25 Dept. of Medicine-Nephrology and Computational Medicine and Bioinformatics University of Michigan, Ann Arbor, MI USA; 26 Dept. of Radiation Oncology, University Hospital Heidelberg, Heidelberg, Germany

**Keywords:** Cancer therapy, Experimental models of disease, Diseases, Cell biology

## Abstract

Radiotherapy remains a mainstay of cancer treatment. However, radiotherapy can also elicit acute and chronic adverse effects, including dermal inflammation and skin fibrosis. A comprehensive understanding of the underlying fibrotic processes remains elusive, and currently, no established treatment options exist. Canonical Wnt signaling has emerged as a significant player in fibrotic conditions. The Dickkopf (DKK) protein family comprises key modulators of Wnt signaling. To define the function of DKK3 in radiation-induced skin damage, we combined complementary in vivo and in vitro approaches, including a 3D human skin model, mice with cell-type-specific Dkk3 deletions, and irradiated human skin specimens. Our study revealed the pivotal role of DKK3 in regulating the response of the skin to radiation, with diminished DKK3 significantly mitigating radiation-induced skin damage. We found that radiation increases DKK3 expression in basal keratinocytes, leading to elevated ROS levels, TGF-β-mediated Wnt activation, epidermal hyperplasia, and subsequent skin fibrosis. Increased keratinocyte expression of DKK3 also drives macrophage polarization toward a CD163^high^CD206^high^ profibrotic M2 phenotype, activating myofibroblasts and leading to fibrosis. Notably, DKK3 deficiency in keratinocytes markedly reduces radiation-induced dermal hyperplasia and fibrosis, identifying DKK3 as a key regulator of cutaneous radiation responses. These findings position DKK3 as a promising upstream modulator of TGF-β signaling for mitigating radiation-induced dermatitis and fibrosis, with potential relevance to other fibrotic diseases.

## Introduction

Radiotherapy is a pivotal local cancer treatment method, yet it can also trigger acute and chronic toxicity in surrounding normal tissues, notably the skin.^1–4^ Skin effects of radiotherapy can range from mild and transient erythema to necrosis, ulcers, and scarring with fibrosis, leading to a reduced quality of life or treatment efficacy for cancer patients.^3,4^

Radiation-induced skin dermatitis and fibrosis stem from a multifaceted interplay of various cellular and molecular factors. These complex interactions between immunologic and nonimmunologic factors involve both immune and structural cells in a dynamic network of cell-to-cell signaling. Immune cells such as macrophages and T cells produce cytokines and growth factors that drive the activation of fibroblasts, leading to excessive deposition of the extracellular matrix, a hallmark of fibrosis. Nonimmune cells, including epithelial cells, also contribute by releasing proinflammatory and profibrotic mediators in response to skin injury, and their dysregulated signaling can help perpetuate inflammation and fibrogenesis.^1–3,5–9^

The repair of skin wounds is a complex, multi-stage, and tightly regulated process in which fibroblasts play central roles, both by orchestrating signaling interactions with other key cell types and by directly contributing to tissue closure and restoration of the injury site.^10^ The transition from acute inflammation to chronic fibrosis is characterized by a shift towards a profibrotic macrophage phenotype.^2,3,11^ These “M2-like” macrophages secrete TGF-β and other chemokines and cytokines and stimulate fibroblast proliferation and differentiation into myofibroblasts, ultimately leading to excessive ECM accumulation and fibrotic tissue. However, a comprehensive understanding of the fibrotic process triggered by radiation remains elusive, and the precise interaction between immunological and nonimmunological factors has yet to be elucidated. Furthermore, no established treatment options exist for managing radiation-induced fibrosis at any anatomical site. Agents targeting TGF-β, PDGF, CTGF,^2,9,12–16^ or CSF1R in macrophages^17^ attenuate radiation-induced fibrosis in preclinical models, partially by reprogramming the immune microenvironment via repolarization of M2-like macrophages.^8,18^ However, for skin fibrosis, only a few interventions, including metformin and adipose-derived stem cells, have shown promise in preclinical modes, likely by inhibiting TGF-β signaling.^19^

The Wnt signaling pathway is a highly conserved regulator of tissue homeostasis, development, and repair.^20^ Wnt signaling is also a central regulator of fibrogenesis across multiple organs.^21^ Excessive activation of the canonical Wnt signaling pathway promotes fibroblast proliferation and myofibroblast differentiation. Persistent or dysregulated Wnt activity stabilizes β-catenin, drives transcription of fibrotic gene programs, and enhances tissue stiffness and inflammatory signaling.^22,23^ Wnt signaling induces the expression of key pro-fibrotic factors, including Connective Tissue Growth Factor (CTGF) and multiple components of the extracellular matrix (ECM), such as collagen I and III.^24,25^ Moreover, crosstalk between Wnt and TGF-β pathways amplifies fibrotic responses, as activation of canonical Wnt signaling has been shown to be required for TGF-β-driven fibrosis.^26^

Dickkopf (DKK) proteins constitute a conserved family of secreted modulators of Wnt signaling. By regulating the activation of Wnt receptors, DKK proteins fine-tune epithelial–mesenchymal communication, stem-cell dynamics, and tissue injury responses, positioning this family as key regulators of both physiological and pathological tissue remodeling.^24,25,27^

Dickkopf-1 (DKK1), DKK2, and DKK4 inhibit canonical Wnt signaling,^27^ whereas DKK3 has divergent biological functions and can either activate or inhibit canonical Wnt signaling depending upon the tissue context.^28,29^ DKK3 plays a significant role in modulating canonical Wnt/β-catenin signaling, with important implications for kidney fibrosis.^30,31^ In cardiac studies, DKK3 was found to prevent dilated cardiomyopathy by activating canonical and inhibiting noncanonical Wnt pathways.^32^ DKK3 is present in normal skin keratinocytes and follicular progenitor cells and is specifically downregulated in keratinocytes by TNF-α, a major inflammatory cytokine.^33^ DKK3 has also been described as an inhibitor of scarring linked to suppressed TGF-β1 signaling in fibroblasts.^34^ These relationships suggested that DKK3 may be involved in the mechanisms underlying inflammatory fibrosing skin disorders, potentially through its role in the Wnt and TGF-β1 signaling pathways and skin cell regulation.

While DKK3 is reduced in diseases characterized by epidermal hyperproliferation,^35^ other studies have demonstrated that DKK3 drives proliferation via canonical Wnt activity and fibrosis.^28,30^ Thus, the role of DKK3 in proliferation and fibrosis remains a topic of debate, and its involvement in radiation-induced tissue responses and toxicity, including fibrosis, remains largely unexplored.

Here, we investigated the role of DKK3 in the skin response to ionizing radiation, with a focus on radiotherapy-induced skin lesions, including hyperproliferation, inflammation, and fibrosis. To dissect the role of DKK3 and Wnt signaling in radiation-induced skin injury, we employed complementary in vivo and in vitro approaches. Targeted single-dose photon irradiation of mouse thoraces and hind limbs was performed in strains carrying cell-type-specific Dkk3 deletions and promoter/reporter transgenes to identify the cellular source and biology driving the phenotype. Mechanistic studies were conducted in human keratinocytes, fibroblasts, and macrophages using single and co-culture systems, including 3D human skin models, with engineered overexpression and reporter constructs. We assessed transcriptional and protein-level responses using bulk gene expression profiling, RT–qPCR, and multiplex cytokine/chemokine assays. Finally, to validate these findings in a clinical context, we analyzed irradiated human skin biopsies by immunohistochemistry, mRNA in situ hybridization, and publicly available single-cell RNA-seq datasets. Our findings demonstrate that the suppression of DKK3 significantly attenuates radiation-induced toxicity, such as hyperplasia and fibrosis. These data reveal that DKK3, which acts as a key regulator, modulates Wnt signaling via TGF-β and influences macrophage activity, thereby orchestrating the response of the skin to radiation.

## Results

### DKK3 promotes radiation-induced hyperplasia and inflammatory and fibrosing skin injury

**Fig. 1:**
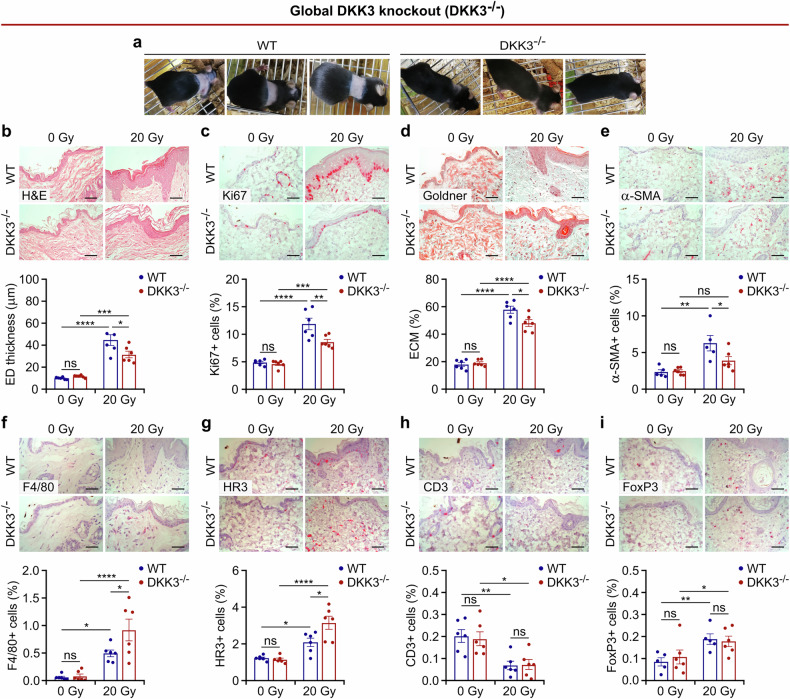
Global DKK3 knockout protects mice from radiation-induced hyperplasia and inflammatory and fibrosing skin injury. Wild-type (WT) and global DKK3 knockout (DKK3^-/-^) mice (*n* = 6/group) were analyzed 4 weeks after 20 Gy thoracic irradiation. Collagen deposition (percentage of extracellular matrix, ECM %) was defined on the basis of the collagen-positive area (green area in Goldner staining)/selected dermal area. The percentage of the indicated marker-positive (Ki67, α-SMA, F4/80, HR3, CD3, and FoxP3) cells was defined on the basis of the positive area (red dots in immunohistochemical staining)/selected area. The epidermis (ED) for Ki67/dermis for other markers was manually selected. **a** DKK3^-/-^ mice were protected from radiation-induced alopecia. **b**–**i** Representative images (top panels) and quantification (bottom panels) of skin sections stained for the indicated markers. Scale bars: 50 μm. The data are presented as the means ± SEMs. Statistical analysis was performed via two-way ANOVA with Tukey’s multiple comparisons test, *P < 0.05, **P < 0.01, ***P < 0.001, ****P < 0.0001

To assess the role of DKK3 in radiation-induced hyperplasia, dermatitis, and skin fibrosis, we irradiated C57BL/N wild-type (WT) and DKK3 global knockout (DKK3^-/-^) mice with a single dose of 20 Gy to parts of the thorax (Fig. 1, supplementary Fig. 1). Independent experiments were performed with the hind limb (supplementary Figs. 2-3). Skin samples were obtained up to three months after irradiation. While WT mice experienced complete in-field alopecia and severe desquamation by 4 weeks after radiation, global DKK3 knockout partly protected the mice from these toxicities (Fig. 1a, supplementary Figs. 2, 3). Histologically, we observed epidermal hyperplasia and extracellular matrix deposition (Goldner, Masson Trichome and α-SMA staining) after radiation, which was markedly reduced in the DKK3^-/-^ mice (Fig. 1b–e, supplementary Fig. 4a–c). Hyperplasia was accompanied by increased expression of the proliferation markers Ki67 and keratins 5 and 10 and the stress marker keratin 16, which were attenuated in the DKK3^-/-^ mice (Fig. 1c, supplementary Fig. 5). Radiotherapy increased macrophage/monocyte (F4/80 and HR3) infiltration and decreased T-cell (CD3) infiltration in WT and DKK3^-/-^ mice (Fig. 1f–h). HR3 staining revealed monocyte/myeloid infiltration of the epidermis and dermis, whereas F4/80 staining (macrophage) was more strongly associated with the dermis. Compared with WT mice, DKK3^-/-^ mice presented greater macrophage/monocyte infiltration, whereas the infiltration of T cells, including Tregs (FoxP3), was not affected by DKK3 status (Fig. 1f–i, supplementary Fig. 2f–h, 3f–h). Overall, our data suggest that DKK3 is involved in radiation-induced hyperplasia and the immune response and functions as a profibrotic factor in radiation-induced skin injury.

### DKK3 expression in keratinocytes in vivo supports dermatitis and profibrotic events

**Fig. 2:**
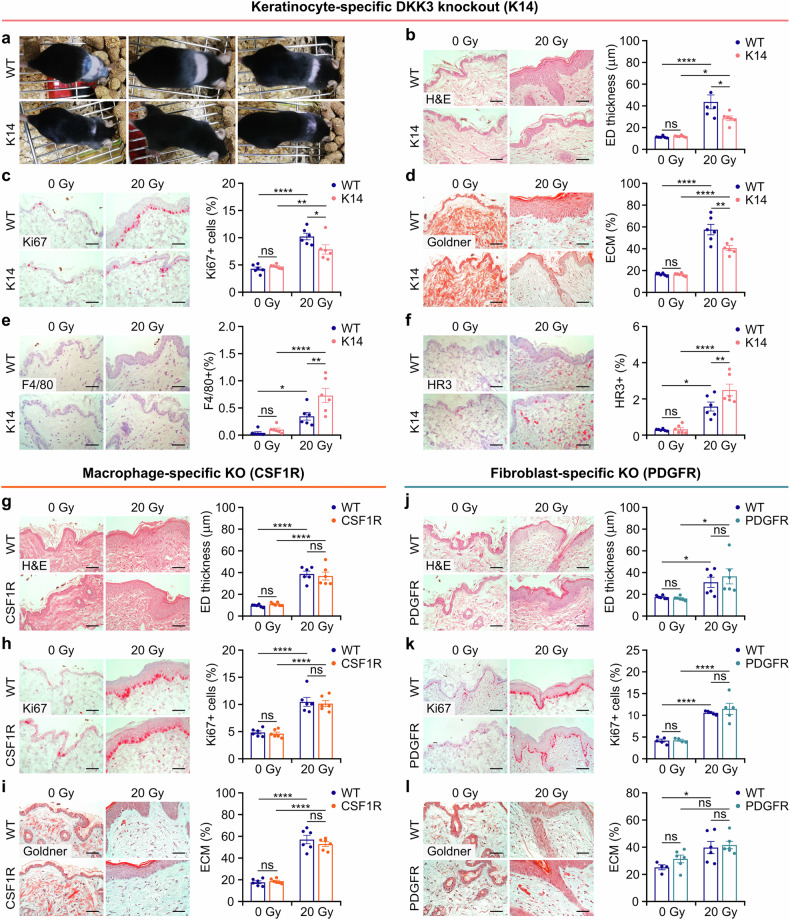
Keratinocyte-specific (K14) DKK3 knockout protects mice from radiation-induced skin toxicity, in contrast to macrophage-specific (CSF1R) and fibroblast-specific (PDGFR) DKK3 knockouts. Wild-type (WT) and cell type-specific DKK3 knockout mice (*n* = 6/group) were analyzed 4 weeks after 20 Gy thoracic irradiation. **a** Under global knockout, keratinocyte-specific DKK3 knockout (K14) mice were protected from radiation-induced alopecia. **b**–**l** Representative images (left panels) and quantification (bottom right) of skin sections stained for the indicated markers. **b**–**f** K14 mice, **g**–**i** mice with macrophage-specific DKK3 knockout (CSF1R) and **j**–**l** mice with fibroblast-specific DKK3 knockout (PDGFR). ED: epidermis. Scale bars: 50 μm. The data are presented as the means ± SEMs. Statistical analysis was performed via two-way ANOVA with Tukey’s multiple comparisons test, *P < 0.05, **P < 0.01, ***P < 0.001, ****P < 0.0001

**Fig. 3:**
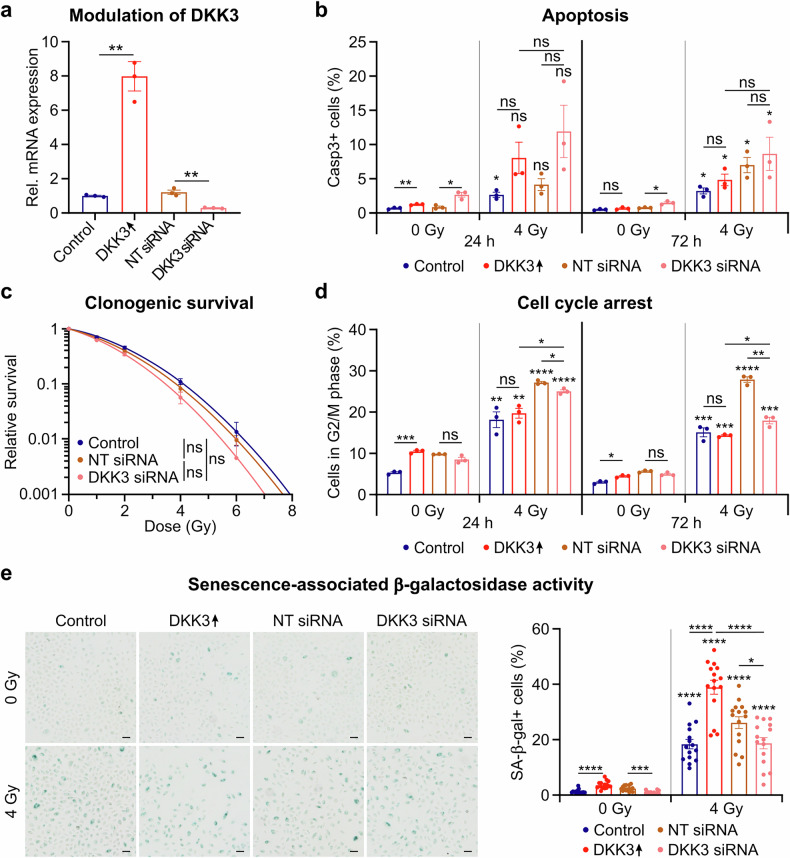
DKK3 status does not significantly modulate intrinsic radiosensitivity but may contribute to radiation-induced senescence. N/TERT 1 keratinocytes were irradiated 24 h after siRNA-mediated DKK3 knockdown (DKK3 siRNA), nontargeting (NT) siRNA, doxycycline-free medium (control) or doxycycline-mediated overexpression of DKK3 (DKK3 ↑ ). **a** Relative DKK3 mRNA expression levels at 24 h after DKK3 modulation. **b** Flow cytometric evaluation of apoptosis-associated caspase-3 activation at 24 h and 72 h after irradiation with 4 Gy (*n* = 3). **c** Clonogenic survival at different radiation doses (*n* = 3). **d** Flow cytometric evaluation of cell cycle arrest in the G2 phase at 24 h and 72 h after irradiation with 4 Gy (*n* = 3) (see also supplementary Fig. 10b). **e** Microscopic evaluation of senescence-associated β-galactosidase (SA-β-gal) activity (*n* = 15 images from 3 replicates). Representative images of stained samples and automated quantification of SA-β-gal-positive cells (%). Scale bars: 50 μm. The data are presented as the means ± SEMs. Statistical analysis was performed via Student’s t test with Holm‒Šidák’s multiple comparison test (**a**, **b**, **d**, **e**) or one-way ANOVA with Tukey’s multiple comparisons test (**c**), *P < 0.05, **P < 0.01, ***P < 0.001, ****P < 0.0001 compared with 0 Gy in the same treatment group or as indicated

To distinguish skin cell type-specific functions of DKK3 in radiation-induced skin fibrosis, we generated cell type-specific knockout mice, including keratinocytes (K14 Cre X Dkk3^fl/fl^,^36^ K14 mice), macrophages/monocytes (CSF1R Cre X Dkk3^fl/fl^,^37^ CSF1R mice), and fibroblasts (PDGFR Cre X Dkk3^fl/fl^,^38^ PDGFR mice). Like global DKK3 knockout, keratinocyte-specific knockout (K14) protected mice from skin toxicity, including alopecia, epidermal hyperplasia, and collagen ECM deposition, in partial thoracic (Fig. 2a–d, supplementary Fig. 6) and hind limb irradiation (supplementary Fig. 7a–d). Moreover, keratinocyte-specific knockout (K14) mice presented greater macrophage/monocyte infiltration than did WT mice (Fig. 2e–f, supplementary Fig. 7e–f). Protective effects were observed at all analyzed time points between 4 weeks (Figs. 2) and 3 months post-radiation (supplementary Fig. 6), suggesting durable protection from skin fibrosis (Fig. 2, supplementary Fig. 6). In contrast, neither macrophage-specific (CSF1R) nor fibroblast-specific (PDGFR) DKK3 knockout protected mice from radiation-induced epidermal hyperplasia or collagen deposition. Instead, these DKK3 knockouts resembled the phenotype observed in WT mice, with no significant difference in immune cell infiltration (Fig. 2g–l, supplementary Fig. 8, 9). Notably, mouse survival did not differ from that of unirradiated controls throughout the 3-month observation period, regardless of DKK3 status or whether the thoracic^12,15^ or hind limb radiation model was used. Hence, these results suggest that DKK3 expression by keratinocytes orchestrates profibrotic events following radiation-induced skin injury.

### DKK3 status does not markedly modulate the intrinsic radiosensitivity of keratinocytes

A potential explanation for the differential responses postradiation may involve the different intrinsic radiosensitivities of keratinocytes associated with DKK3 status. The hallmark of radiosensitivity is radiation-induced DNA damage and repair.^1,2,39^ To explore whether DKK3 knockout confers protection against skin damage by enhancing radioresistance, we compared human N/TERT-1 keratinocytes with downregulated or overexpressed DKK3 (Fig. 3a, supplementary Fig. 10). DKK3 modulation in keratinocytes did not significantly influence radiation-induced apoptosis (Fig. 3b), radiation-induced reduction in clonogenic survival (Fig. 3c), or radiation-induced reduction in proliferation (supplementary Fig. 10a). Accordingly, the DNA double-strand break (DSB) marker γH2AX did not significantly differ in terms of DNA damage induction or repair dynamics between DKK3 knockdown, overexpression or unstimulated expression (supplementary Fig. 10c). Hence, the DKK3 expression status did not significantly affect the intrinsic radiosensitivity of keratinocytes. However, DKK3 overexpression increased cell cycle arrest, which was reduced by DKK3 knockout (Fig. 3d, supplementary Fig. 10b). Additionally, radiation induced senescence-associated β-galactosidase (SA-β-gal) activity, which increased after DKK3 overexpression (Fig. 3e), suggesting that DKK3 may contribute to radiation-induced senescence.

### Radiation-induced DKK3 expression is associated with canonical Wnt activation in basal keratinocytes

**Fig. 4:**
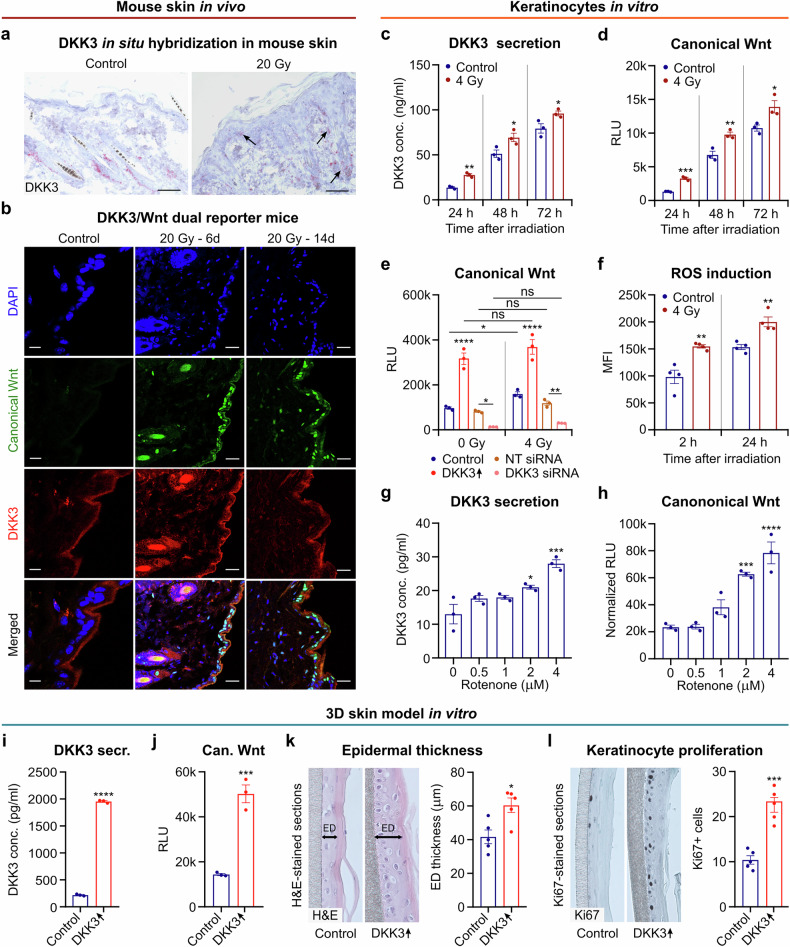
Radiation increases DKK3 expression and canonical Wnt activity in vivo and in vitro via ROS. **a** Representative in situ hybridization images of DKK3-positive cells in thoracic skin sections of irradiated (20 Gy) and nonirradiated (control) wild-type mice at 4 weeks post-irradiation (*n* = 5 skin sections/mouse from *n* = 5 mice/group). Scale bars: 25 μm. **b** Representative immunofluorescence images of thoracic skin sections from DKK3/canonical Wnt dual reporter mice at 0 days (control, *n* = 2), 6 days (*n* = 2), and 14 days (*n* = 2) postirradiation with 20 Gy. Canonical Wnt activity is represented by GFP (green), and DKK3 expression is represented by the m-Cherry signal (red). Nuclei are stained with DAPI (blue). Scale bars: 50 μm. N/TERT-1 keratinocytes with the Wnt reporter were irradiated in vitro with 4 Gy. **c** DKK3 levels (ELISA) and **d** canonical Wnt activity (*Gaussia* luciferase assay) in culture media at different time points postirradiation. RLU: relative light units. **e** Canonical Wnt activity in the culture media of Wnt-reporter keratinocytes transfected with DKK3 siRNA, nontargeting (NT) siRNA or mock-transfected controls at 24 h postirradiation with 4 Gy. **f** ROS levels (H2DCFDA assay) in Wnt-reporter keratinocytes at 2 and 24 h postirradiation with 4 Gy. MFI: mean fluorescence intensity. Wnt-reporter keratinocytes were treated with different concentrations of the chemical ROS inducer rotenone. **g** DKK3 levels (ELISA) and **h** canonical Wnt activity (*Gaussia* luciferase assay) in the media at 12 h after treatment. **i**–**l** 3D skin model of Dox-induced DKK3-overexpressing (DKK3 ↑ ) Wnt-reporter keratinocytes and unstimulated controls. **I** DKK3 levels (ELISA) and **j** canonical Wnt activity (*Gaussia* luciferase assay*)* in culture media at 72 h after Dox induction. **k** Representative image of H&E-stained 3D skin sections and quantification of epidermal (ED) thickness. **l** Representative image and quantification of Ki67-stained sections. The data are presented as the means ± SEMs. Statistical analysis was performed via Student’s *t* test (**a**-**d**, **f**, **i**-**l**), one-way ANOVA with Tukey’s multiple comparisons test (**g**, **h**) or two-way ANOVA with Tukey’s multiple comparisons test (**e**), *P < 0.05, **P < 0.01, ***P < 0.001 compared with the control or as indicated

We next showed via in situ hybridization that radiation directly increased DKK3 expression in mouse skin in vivo (Fig. 4a). DKK3 was upregulated in basal keratinocytes, whereas DKK3-positive cells in the outer root sheath of hair follicles remained unchanged. Additionally, DKK3-expressing myofibroblasts were observed in the dermis at 4 weeks post-irradiation. Immunohistochemistry confirmed that radiation increased DKK3 expression in keratinocytes, mostly in the basal layer and partly in the granular layer, and in dermal myofibroblasts (supplementary Fig. 11), as did constitutive DKK3 expression in the outer bulge cells of hair follicles.^40^ Since DKK3 regulates canonical Wnt signaling, we further characterized the spatiotemporal expression of DKK3 and canonical Wnt activity. We generated dual-reporter mice by crossing two preestablished transgenic mouse lines with a DKK3 promoter-driven m-Cherry reporter and a canonical Wnt-dependent GFP reporter.^30,41^ In these dual-reporter mice, irradiation strongly increased DKK3 expression and canonical Wnt activity in the basal keratinocyte layer at 6 and 14 days after irradiation (Fig. 4b). To model DKK3-driven canonical Wnt activity in vitro, the N/TERT-1 keratinocyte cell line was stably transfected with a plasmid encoding a synthetic TCF-based promoter driving a secreted *Gaussia* luciferase reporter gene.^42^ We found that irradiation markedly increased both the DKK3 level and the Wnt reporter signal in the medium at 24, 48, and 72 hours post-treatment (Fig. 4c, d). Conversely, DKK3 knockdown abrogated the basal and radiation-induced increase in canonical Wnt activity (Fig. 4e). These results suggest that DKK3 expression in keratinocytes plays a crucial role in radiation-induced Wnt activity.

### Radiation-induced ROS increase DKK3 expression and Wnt activity in keratinocytes

Reactive oxygen species (ROS) are important mediators of radiation effects.^1–3^ We therefore investigated ROS as potential mediators of radiation-induced DKK3 expression. First, we confirmed that irradiation increased ROS levels in keratinocytes via the H2DCFDA assay (Fig. 4f). Then, we showed that ROS are sufficient to drive DKK3 expression and canonical Wnt signaling without radiation when the chemical ROS inducer rotenone is used (Fig. 4g, h). Using a human 3D skin model^43^ based on N/TERT-1 canonical Wnt reporter/Dox-inducible DKK3 cells, we confirmed that increased DKK3 expression leads to increased canonical Wnt activity in the absence of radiation or a ROS inducer (Fig. 4i, j). DKK3 overexpression in this 3D model also significantly increased keratinocyte proliferation, as measured by the thickness of the keratinocyte layer, which is a surrogate for epidermal hyperplasia, and the proliferation markers Ki67 (Fig. 4k, l) and PCNA (supplementary Fig. 12). These results parallel the epidermal thickness measurements and Ki67 staining results in mice (Figs. 1b, c, 2b, c; supplementary Fig. 2b, c, 3b, c). Hence, our combined 2D, 3D skin model and in vivo mouse results indicate that increased DKK3 expression in keratinocytes results in increased canonical Wnt activity and subsequent epidermal thickening.

### DKK3 expression by keratinocytes activates canonical Wnt activity via TGF-β

**Fig. 5:**
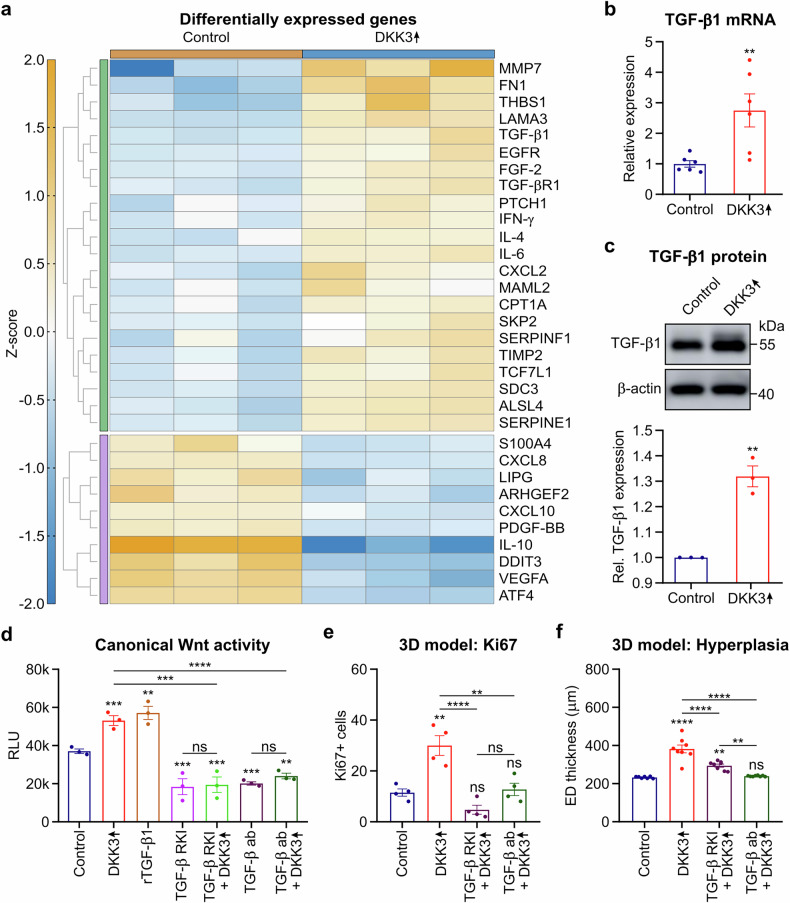
DKK3 activates canonical Wnt activity via TGF-β1 in keratinocytes. **a**–**f** N/TERT-1 keratinocytes with Dox-inducible DKK3 overexpression and a canonical Wnt reporter were stimulated with Dox (DKK3 ↑ ) for 24 h and compared with unstimulated controls. **a** Heatmap showing gene expression analysis (nCounter) of substantially and significantly differentially expressed genes (orange indicates upregulation, and blue indicates downregulation by DKK3↑ versus the control; *n* = 3 biological replicates, >1.4-fold change, *p* < 0.05). **b** TGF-β1 mRNA levels (RT‒qPCR). **c** TGF-β1 protein levels (western blot). **d** Cells were treated with TGF-β1 protein (rTGF-β1), a small molecule inhibitor of the TGF-β receptor 1 and 2 kinase (TGF-β RKI, LY2109761), or a TGF-β antibody (TGF-β ab) 24 h after Dox-induced DKK3 overexpression. Canonical Wnt activity was measured 24 h later in the culture medium (*Gaussia* luciferase assay). RLU: relative light units. **e**, **f** 3D-skin models of N/TERT-1 keratinocytes with Dox-induced or unstimulated DKK3 overexpression were treated with TGF-β RKI or TGF-β. **e** Quantification of Ki67-stained sections and **f** epidermal thickness in H&E-stained sections at 72 h after TGF-β inhibition. The data are presented as the means ± SEMs. Statistical analysis was performed via Student’s t test (**b**, **c**) or one-way ANOVA with Tukey’s multiple comparisons test (**d**–**f**), *P < 0.05, **P < 0.01, ***P < 0.001, ****P < 0.0001 compared with the control or as indicated

TGF-β is a key mediator of various types of fibrosis, including radiation-induced fibrosis.^1–3,8,39^ Immunostaining for mouse TGF-β revealed increased basal keratinocyte expression 4 weeks following 20 Gy irradiation in WT animals but little or no increase in TGF-β expression in keratinocyte (K14)-specific knockout animals (supplementary Fig. 13). Crosstalk between TGF-β and canonical Wnt signaling pathways has been described in fibrogenesis.^26^ We hypothesized that TGF-β may act as a signaling link between DKK3 and canonical Wnt pathway activation. First, we assessed select transcriptomic alterations following DKK3 overexpression in keratinocytes via the nCounter system (Fig. 5a) and validated selected genes via RT‒qPCR. DKK3 upregulation was verified by RT‒qPCR, as nCounter, a human fibrosis-specific panel, does not contain a probe for DKK3 (Fig. 3a). TGF-β1 (p = 0.02) and TGF-β1R1 (p = 0.006) were significantly upregulated due to DKK3 overexpression (Fig. 5a–c; supplementary Table 1). Stimulation with recombinant TGF-β1 alone activated canonical Wnt pathway signaling in the reporter keratinocytes (Fig. 5d). In addition, blocking TGF-β signaling in the context of DKK3 overexpression via the small-molecule TGF-βR-1/2 kinase inhibitor LY2109761 (TGF-β RKI)^44^ or a monoclonal antibody against TGF-β 1, 2, and 3 (TGF-β ab) abrogated DKK3-associated canonical Wnt signaling (Fig. 5d). This result was confirmed in the 3D skin model, where TGF-β RKI or TGF-β ab blocked DKK3-driven keratinocyte proliferation (Ki67) and hyperplasia (Fig. 5e, f). Thus, DKK3 appears to drive canonical Wnt activity, accompanied by epidermal hyperplasia, through crosstalk with the TGF-β pathway.

**Fig. 6:**
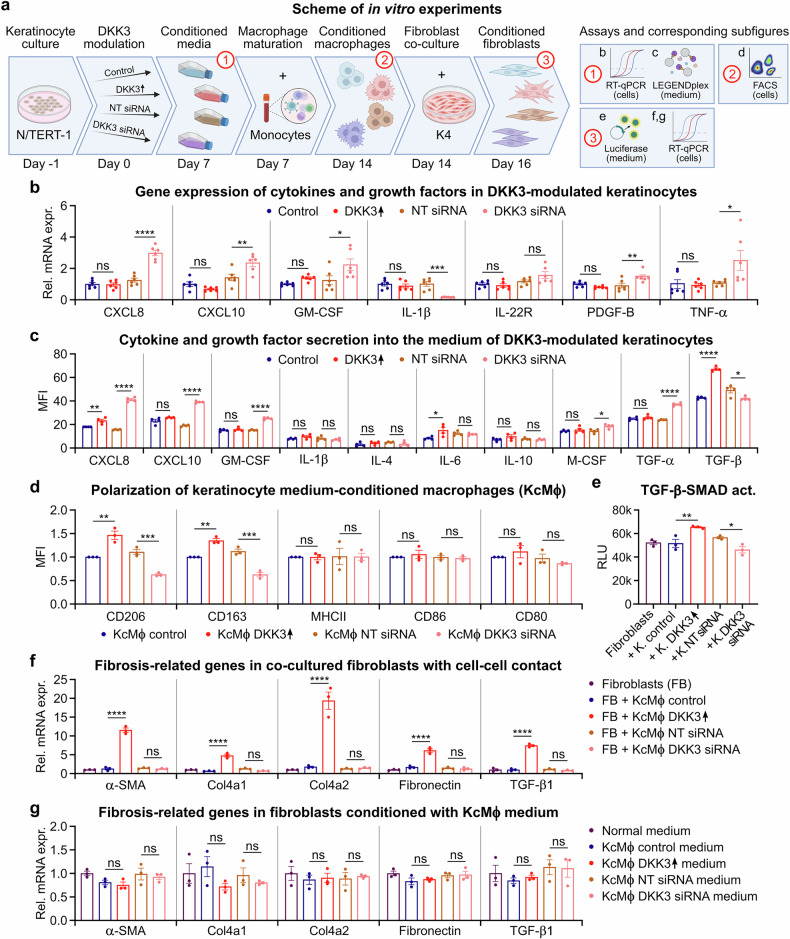
DKK3 induction in keratinocytes triggers a broad profibrotic signaling cascade leading to macrophage polarization and myofibroblast activation in vitro. **a** DKK3 was knocked down (DKK3 siRNA, NT siRNA refers to nontargeting siRNA controls), overexpressed (DKK3 ↑ ) or left unmodulated (control) in N/TERT keratinocytes. After 7 days, cytokine and growth factor expression in the cells was quantified via RT‒qPCR. The conditioned media were harvested for multiplex antibody-based protein profiling and for the maturation of macrophages from freshly isolated monocytes from human PBMCs for another 7 days. Keratinocyte-conditioned macrophages (KcMф) were characterized on the basis of surface marker expression. K4 fibroblasts were either cocultured with KcMф or cultured in KcMф supernatants for only 2 days. The expression of fibrosis-related genes was evaluated in these conditioned fibroblasts. **b** Cytokine and growth factor expression (RT‒qPCR) and **c** multiplex antibody-based protein secretion in DKK3-modulated keratinocytes. **d** The M2-associated surface markers CD206 and CD163 (FCM) were increased in macrophages conditioned with media from DKK3↑ keratinocytes (KcMф DKK3 ↑ ) and decreased in macrophages conditioned with media from DKK3-knockdown keratinocytes (KcMф DKK3 siRNA). **e** TGF-β–SMAD pathway activity (*Gaussia* luciferase assay) in K4 fibroblasts after coculture with conditioned macrophages. **f** Fibrosis-related gene expression (RT‒qPCR) in K4 fibroblasts directly cocultured (cell‒cell contact) with conditioned macrophages. Only macrophages cultured with DKK3-overexpressing keratinocytes (KcMф DKK3 ↑ ), but not those cultured with DKK3-knockdown keratinocytes (KcMф DKK3 siRNA), exhibited increased expression of profibrotic genes in K4 fibroblasts. **g** Fibrosis-related gene expression (RT‒qPCR) in K4 fibroblasts was unaffected by KcMф supernatants (no cell‒cell contact). The data are presented as the means ± SEMs. Statistical analysis was performed via one-way ANOVA with Tukey’s multiple comparisons test, *P < 0.05, **P < 0.01, ***P < 0.001, ****P < 0.0001 compared with the control or as indicated. Figure 6 was partially created in BioRender. Huber, P. (2025) https://BioRender.com/w3iggj5

Next, we used nCounter gene expression analysis coupled with RT‒qPCR and multiplex antibody-based protein profiling to further analyze cytokine and chemokine secretion. We found that upregulating DKK3 expression in keratinocytes altered the expression of immune-regulatory cytokines and fibrosis-related genes and pathways (Figs. 5a, 6b, c; supplementary Tables 1, 2).

DKK3-overexpressing keratinocytes upregulated the expression of genes associated with extracellular matrix (EMC) remodeling (MMP-7, TIMP2, LAMA3, FN1, THBS1, EGFR, PTCH1, and Serpinf1), Wnt signaling (TCF7L1), and Th2 differentiation (IL-4) (Fig. 5a, supplementary Table 1). Pathway enrichment analysis revealed that the genes differentially expressed (irrespective of their up- or downregulation) belong to pathways linked to “profibrotic” phenotype development or “associated with fibrosis”, including T-helper 2 differentiation and epithelial-to-mesenchymal transition (EMT). The MAPK, PI3K-AKT and Notch pathways are associated with cell stress, whereas canonical Wnt signaling is a major driver of EMT and skin fibrosis, acting both independently and in synergy with TGF-β (supplementary Table 2).

In line with the increased rate of cellular senescence (Fig. 3e), DKK3 overexpression increased the expression of senescence-associated secretory phenotype (SASP) components^2,45–47^ including IL-6, TGF-β, CXCL2, and MMP7, while DKK3 knockdown decreased IL-1β expression (Figs. 5a, 6b, c; supplementary Table 1).

Additionally, DKK3 knockdown increased the expression and secretion of the key myeloid differentiation cytokines GM-CSF and M-CSF and other factors involved in acute inflammation, antifibrotic effects and cellular regulation (PDGF-B, IL-22R, TGF-α, and TNF-α) (Fig. 6b, c). This finding suggests that upregulated DKK3 can significantly impact the keratinocyte secretome, resulting in profibrotic signaling, including potential downstream effects on profibrotic immune polarization.

### Keratinocyte-derived DKK3 promotes profibrotic macrophage polarization

Macrophages are important regulators of the ECM composition^2,3^ and present highly plastic phenotypes, with markers such as CD163 and CD206 indicating a profibrotic M2-like polarization status.^48^ Our global and keratinocyte-specific DKK3 knockout mice were protected from radiation injury, despite increased monocyte/macrophage infiltration (Figs. 1f, g, 2e, f; supplementary Figs. 2f, g, 3f, g). This finding suggested that the activation status of the immune infiltrate within the tissue microenvironment dictates the fate of tissue fibrosis. We thus hypothesized that the upregulation of immune-related factors in DKK3-overexpressing human keratinocytes might promote profibrotic macrophage polarization, while DKK3 knockdown would reduce these effects. This takes on additional significance given the enhanced infiltration of monocyte/myeloid cells (HR3 stain) observed in the epidermis and dermis following radiation treatment, thus positioning them to respond to the keratinocyte secretome (Figs. 1f, g, 2e, f, supplementary Fig. 2f, g, 3f, g).

To investigate this hypothesis in vitro, we investigated changes in macrophage polarization in response to macrophage–keratinocyte interactions. We incubated primary human blood monocytes in human keratinocyte (N/TERT-1)-conditioned media after modulating DKK3 expression (Fig. 6a). We found that macrophages conditioned in media from DKK3-overexpressing keratinocytes presented increased expression of CD206 and CD163, whereas macrophages conditioned in media from DKK3-knockdown keratinocytes presented decreased expression of CD206 and CD163 (Fig. 6d). In contrast, MHCII, CD80 and CD86 surface marker expression in macrophages was not significantly altered by DKK3 modulation in keratinocytes. These data support the notion that high DKK3 expression in keratinocytes is associated with a profibrotic M2-like macrophage phenotype.

We next investigated the interaction of macrophages with human fibroblasts because myofibroblasts are the major drivers of fibrotic processes.^48,49^ We prepared keratinocyte-conditioned macrophages (KcMф) as previously described and cocultured them with human dermal fibroblasts (K4) stably transfected with a TGF-β pathway-dependent *Gaussia* luciferase reporter under the control of a SMAD promoter (validation in supplementary Fig. 18).^42^ KcMф derived from DKK3-overexpressing keratinocytes activated TGF-β–SMAD pathway signaling (Fig. 6e) and increased the mRNA levels of α-SMA, Collagen 4a1, Collagen 4a2, Fibronectin (FN1) and TGF-β1 (Fig. 6f) in the fibroblast reporter cell line. Conversely, macrophages cultured with media from DKK3-knockdown keratinocytes or unconditioned macrophages did not upregulate the expression of these profibrotic genes in fibroblasts. Notably, these profibrotic effects in the macrophage‒fibroblast coculture were observed only under direct cell‒cell contact; KcMф-conditioned medium alone was not sufficient to stimulate fibroblasts (Fig. 6g).

We also conducted a series of experiments using irradiated and nonirradiated DKK3-modulated keratinocytes (supplementary Fig. 14). Compared with their nonirradiated counterparts, irradiated keratinocytes without DKK3 modulation could also promote macrophage polarization toward the CD206^high^ and CD163^high^ phenotypes. Notably, the expression of fibrosis-related genes—α-SMA, collagen 4a2, and TGF-β1—was significantly upregulated in the fibroblasts after direct coculture with KcMф but not in the fibroblasts in KcMф-conditioned media, confirming the requirement for direct cell‒cell contact between fibroblasts and macrophages. Furthermore, in the groups of irradiated DKK3-overexpressing or DKK3-deficient keratinocytes, macrophage polarization and fibrosis gene expression largely mirrored the unirradiated results. Radiation thus partially recapitulates the effects of DKK3 overexpression in keratinocytes, particularly in driving macrophage-mediated fibrotic signaling.

**Fig. 7:**
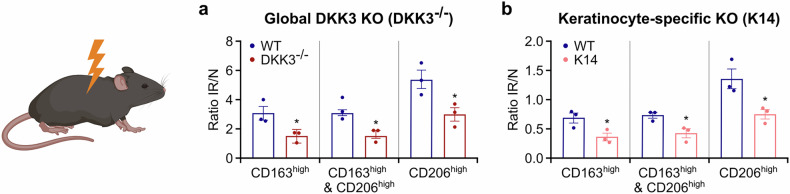
Keratinocyte DKK3 promotes profibrotic macrophage polarization in vivo. **a** Wild-type (WT), DKK3 global knockout (DKK3^-/-^), and keratinocyte-specific DKK3 knockout (K14 Cre X Dkk3^fl/fl^) mice were subjected to 20 Gy thoracic irradiation. Four weeks post-irradiation, skin cells were isolated from irradiated (IR) and nonirradiated (N) skin regions and analyzed by flow cytometry, gating for CD163high and CD206high macrophages (the gating strategy is shown in supplementary Fig. 15). The ratio of the number of macrophages with the indicated surface marker expression in irradiated versus nonirradiated skin (IR/N) is shown. **a** Polarization markers in DKK3 global knockout (DKK3^-/-^) mice compared with WT mice. **b** Keratinocyte-specific DKK3 knockout (K14) mice compared with WT mice. Different mice presented different frequency distributions of cell subsets before treatment. Ratios of cell subset frequencies between the nontreated (NR) and treatment (IR) groups were used to allow an efficient summation of different mouse data. The data are presented as the means ± SEMs. Statistical analysis was performed via Student’s *t* test, *P < 0.05 compared with WT. Figure 7 was created in BioRender. Huber, P. (2025) https://BioRender.com/jpaliv7

In line with these in vitro data, both global and keratinocyte-specific DKK3 knockout mice presented markedly decreased proportions of radiation-induced CD206^high^, CD163^high^, and CD163^high^ & CD206^high^ macrophages in their skin 4 weeks after radiotherapy (Fig. 7). Taken together, our findings suggest that DKK3 expression in keratinocytes initiates a broader profibrotic signaling cascade that is essential for the radiotherapy-induced profibrotic reprogramming of macrophage phenotypes.

### DKK3 expression is increased in human skin after irradiation and other fibrosing skin diseases

**Fig. 8:**
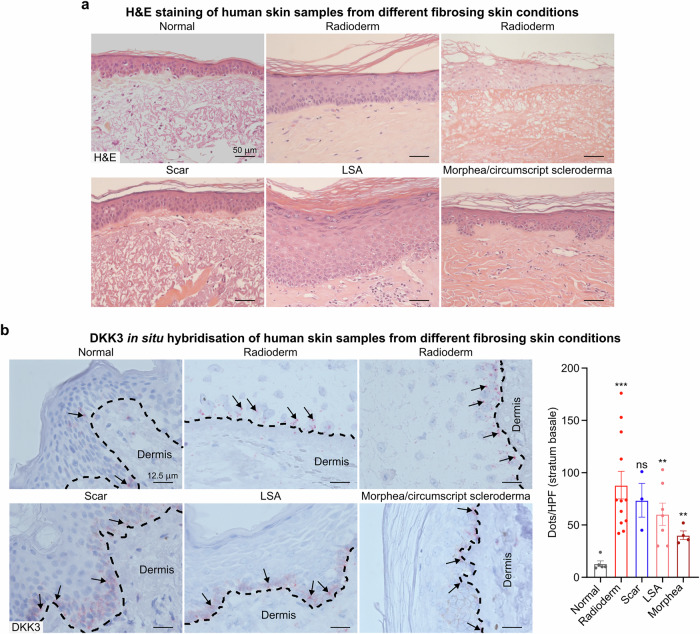
DKK3 in patient-derived human skin samples after irradiation and other fibrosing insults. Patient-derived skin biopsies from normal skin and different fibrosing conditions (chronic radiation dermatitis, scaring, Lichen sclerosus et atrophicus (LSA) and morphea/circumscript scleroderma) were H&E stained (**a**) and hybridized in situ for the presence of DKK3 mRNA (reddish dots, see quantification in the right panel) (**b**). Compared with normal skin, diseased samples presented with epithelial hyperplasia with inflammatory infiltration (**a**). Normal skin basal keratinocytes are barely stained for DKK3 (black arrows) and sometimes for dermal cells, but enhanced DKK3 expression is present in basal keratinocytes (the layer marked with dashed lines) after irradiation (radioderm), in scarred tissue and in the fibrosing skin of LSA and morphea patients (black arrows highlight some examples) (**b**). The quantification of DKK3-positive dots per high-power field (HPF) in basal keratinocytes revealed a significant increase in the incidence of radiation dermatitis, LSA fibrosis and morphea. The data are presented as the means ± SEMs. Statistical analysis was performed via Student’s *t* test with unequal variances, **P < 0.01, ***P < 0.001 compared with the control

These experimental findings strongly suggest a central role for increased basal keratinocyte DKK3 expression in driving dermal hyperplasia, myeloid polarization, and fibrogenesis. To validate our experimental findings, we next investigated human skin samples from patients after radiotherapy for cancer and patients with other fibrosing skin diseases. Epidermal hyperplasia accompanied by tissue fibrosis was observed in the irradiated samples, as evidenced by H&E staining (Fig. 8a). DKK3 mRNA expression was evaluated via in situ hybridization across a series of human skin biopsy samples representing disease entities characterized by fibrosis (Fig. 8b). Examples of chronic radiation dermatitis, skin scarring, Lichen sclerosus et atrophicus (LSA) and morphea (localized scleroderma) were evaluated. Compared with that in normal control skin, increased expression of DKK3 mRNA within the basal keratinocyte layer was detected in each of the pathological conditions, with the highest expression occurring in samples from individuals with chronic radiation dermatitis (Fig. 8c). Furthermore, immunohistochemical staining in an independent series of patients receiving radiotherapy for cancer confirmed that radiation increased DKK3 expression in human skin (supplementary Fig. 19). In line with these findings, analysis of publicly available single-cell sequencing data from irradiated human skin revealed increased DKK3 expression in keratinocytes (supplementary Fig. 20, 21).^50^ When all cells were analyzed collectively, global DKK3 expression increased (*p* < 0.001), indicating a systemic response to radiation. Keratinocyte subpopulations exhibited divergent shifts: the number of basal undifferentiated keratinocytes increased postradiation (p < 0.001), whereas the number of terminally differentiated epidermal granular keratinocytes decreased (*p* < 0.01). Pooled keratinocyte analysis confirmed that DKK3 was elevated (*p* < 0.001), with basal keratinocytes dominating the postradiation keratinocyte compartment. Further expression analysis of keratinocytes revealed that irradiation significantly upregulated genes associated with fibrosis and Wnt signaling, including TGF-β1, COL1A1, fibronectin (FN1), vimentin (VIM), TCF4, VEGFA, and ZEB1 (supplementary Fig. 21b).^25,26^ Stratification of keratinocytes into DKK3^high^ and DKK3^low^ subpopulations revealed that the expression of fibrosis-associated genes (TGF-β1 and COL1A1) and key downstream targets of Wnt signaling (FN1, VIM, ZEB1, JUN, TCF7,3,4, BCL9, Myc, CCND1, NKD1, MMP7, BIRC5, and VEGFA) was consistently elevated in the DKK3^high^ group (supplementary Fig. 21c). These findings suggest coordinated regulation and a functional role for Wnt-associated DKK3 in the cellular response to radiation. Gene set enrichment analysis further revealed a radiation-induced phenotypic shift in keratinocytes toward enhanced migratory capacity and a profibrotic state characterized by extracellular matrix (ECM) reorganization (supplementary Fig. 21a). Collectively, these signaling findings demonstrate that radiation triggers global DKK3 upregulation concurrently with keratinocyte reprogramming, which drives skin fibrogenesis.

## Discussion

**Fig. 9:**
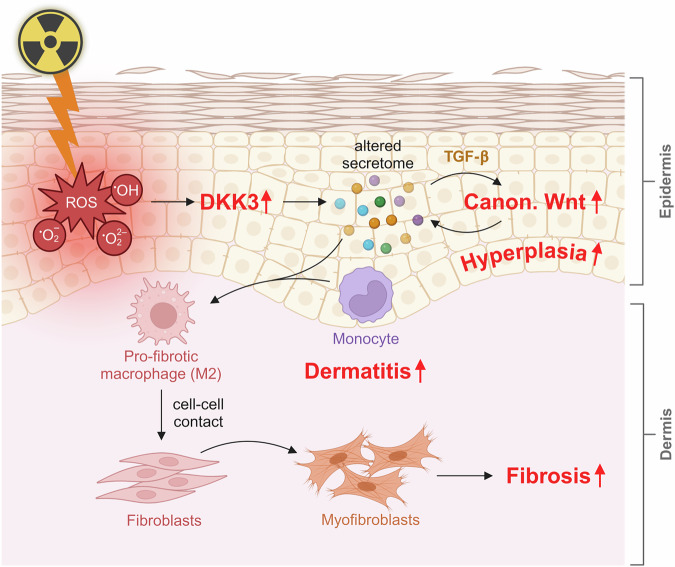
Graphical summary: DKK3 in keratinocytes orchestrates radiation-induced skin hyperplasia, dermatitis, and fibrosis. Radiation-induced reactive oxygen species (ROS) increase DKK3 expression in keratinocytes, which subsequently activates canonical Wnt signaling through autocrine TGF-β signaling. Elevated DKK3 levels in keratinocytes drive hyperproliferation and hyperplasia, promoting the polarization of macrophages toward a profibrotic phenotype. These polarized macrophages, in turn, upregulate fibrosis-associated factors in myofibroblasts, contributing to the fibrotic response. Figure 9 was partially created in BioRender. Huber, P. (2025) https://BioRender.com/19y6inq

This study identified DKK3 as a key regulator of radiation-induced skin effects. Our findings reveal that DKK3 deficiency in basal keratinocytes mitigates radiation-induced epidermal hyperplasia, dermatitis, and fibrosis. Radiation exposure triggers DKK3 expression, leading to increased TGF-β production and the activation of canonical Wnt signaling in keratinocytes (Fig. 9). These changes are associated with epithelial hyperplasia, dermatitis and fibrosis. Conversely, the absence of DKK3 in keratinocytes results in reduced epithelial hyperplasia and decreased profibrotic polarization of inflammatory macrophages in the skin (Figs. 2, 7; supplementary Figs. 2–4). These findings suggest that keratinocyte-derived DKK3 alters the inflammatory microenvironment in the skin. Our results highlight the crucial role of DKK3 in the skin response to radiation injury. This study proposes DKK3 as a potential novel target for treating radiation-induced dermatitis and skin fibrosis.

DKK3 is expressed in many human cell types and has been reported to be downregulated in diseases characterized by epithelial hyperplasia, including skin disorders.^35^ However, our findings challenge this previously suggested negative association between DKK3 and epidermal proliferation. We consistently observed that radiation induces increased DKK3 expression in keratinocytes and that this increased DKK3 expression leads to epithelial activation and hyperplasia. In line with our preclinical data, we validated our findings in human skin samples, which revealed increased expression of DKK3 mRNA in the basal keratinocyte layer in patients with chronic radiation dermatitis after radiotherapy for cancer (Fig. 8b, supplementary Figs. 19, 20, 21). In addition, a similar increase in DKK3 mRNA expression was also observed in patients with fibrotic scarring and in the autoimmune settings of Lichen sclerosus et atrophicus (LSA) and morphea (localized scleroderma) (Fig. 8b). Furthermore, the upregulation of DKK3, TGF-β1, COL1A1, FN1, VIM, TCF4, VEGFA, and ZEB1 mRNA in keratinocytes was independently confirmed when we analyzed a published scRNA sequencing dataset from the skin samples of patients after accidental high-dose radiation exposure.^50^ Fibrosis-related genes (TGF-β1, COL1A1) and key downstream targets of Wnt signaling (FN1, VIM, ZEB1, JUN, TCF7, 3, 4, BCL9, Myc, CCND1, NKD1, MMP7, BIRC5, and VEGFA) were also upregulated in the DKK3^high^ vs. DKK3^low^ keratinocyte subpopulation, suggesting coordinated regulation and a functional role for Wnt-associated DKK3 in the cellular response to radiation. Gene set enrichment analysis further revealed a radiation-induced phenotypic shift toward enhanced migratory capacity and a profibrotic state characterized by extracellular matrix (ECM) reorganization. Recent reports have highlighted the central role of canonical Wnt signaling in driving fibrotic processes, often in close interaction with TGF-β pathway activation.^51–53^ These results suggest a more complex role for DKK3 in epidermal regulation, particularly in the context of radiation-induced skin responses (Fig. 9). In an in vivo mouse model, DKK3 expression increased in basal keratinocytes early after radiation, suggesting that basal keratinocytes represent an important cellular source of DKK3 following radiation damage (Fig. 4a, b). It is plausible that DKK3 has diverse and potentially contradictory functions in hyperproliferation and fibrosis progression. DKK3 expression and its downstream effects may be dependent on the nature of the injury or the specific cellular context, similar to what is known about the pleiotropic nature of TGF-β.^39,54^ We validated our findings through targeted depletion of DKK3 in relevant skin cells in murine models of radiation toxicity and through in vitro studies in human cell lines (Figs. 2, 4). Radiation increased DKK3 and Wnt reporter signaling, whereas DKK3 knockdown abrogated the basal and radiation-induced increases in canonical Wnt activity (Fig. 4). These findings suggest that functional DKK3 may be necessary for radiation-induced canonical Wnt pathway signaling. Overall, we identified DKK3 expression in keratinocytes as a key mechanism in radiation-induced dermal hyperplasia and fibrosis.

Radiation-induced reactive oxygen species (ROS) can cause a wide spectrum of biological effects, including dermatitis and fibrosis.^1–3^ Our data suggest that ROS contribute to DKK3 upregulation in keratinocytes following radiation treatment, thus bridging the signaling gap between the physical effects of ionizing radiation and subsequent fibrotic biology (Fig. 4). This connection, which was previously unexplored, highlights the pivotal role of ROS-induced DKK3 expression in epithelial cells as the initiating event in radiation-induced fibrosis. DKK3 may thus exacerbate chronic toxicity by promoting inflammatory fibrosis through secondary pathways. Importantly, DKK3 expression had only a minor effect on the intrinsic radiosensitivity of keratinocytes in vitro, as determined by clonogenic survival, DNA repair, and apoptosis assays. Therefore, the reduced hyperplasia and dermal fibrosis observed in DKK3-deficient mice following radiation are unlikely to be due to altered keratinocyte radiosensitivity. Instead, alternative mechanisms, such as radiation-induced DKK3 upregulation in stressed basal keratinocytes, activation of Wnt signaling, and subsequent immunomodulatory effects, are likely responsible. Our findings indicate that overexpression of DKK3 in keratinocytes leads to increased expression of TGF-β, subsequently activating canonical Wnt pathway activity in keratinocytes (Figs. 5, 6). Blocking the TGF-β pathway in keratinocytes eliminated DKK3-induced canonical Wnt pathway activation (Fig. 5d). These results align with previous reports that TGF-β and canonical Wnt signaling cross-talk in radiation-dependent^55^ and radiation-independent^26,56^ models of fibroblast activation and ECM accumulation. While TGF-β signaling is strongly associated with tissue fibrosis, it is also important in regulating multiple signaling pathways, including metabolism, epigenetics, aging, and other important cellular processes.^39,54^ Strategies targeting the TGF-β pathway to treat radiation injury have shown positive effects, but the timing and specificity of these agents are complex enough to avoid disrupting the normal function of TGF-β in immune regulation and tissue repair.^3^ Suppressing DKK3 could thus be a more specific antifibrotic treatment approach.

Global and keratinocyte-specific DKK3 knockout mice were protected from radiation damage, whereas macrophage- and fibroblast-specific DKK3 knockout mice exhibited a phenotype similar to that of WT mice (Fig. 2, supplementary Fig. 6, 8). Notably, the protected global and keratinocyte-specific DKK3 knockout mice presented increased infiltration of monocytes and macrophages into the skin, which surpassed the elevated infiltration observed in the irradiated WT animals (Figs. 1f, g, 2e, f; supplementary Figs. 2f, g, 3f, g). These findings appear counterintuitive given the presumed role of inflammatory infiltrates as drivers of chronic fibrosis. However, macrophages are heterogeneous cells with considerable plasticity and diversity that are thought to influence inflammatory fibrosis by shifting between antifibrotic and profibrotic phenotypes on the basis of signals present in the specific tissue environment.^48^ Furthermore, radiation immune responses and macrophage polarization depend significantly on the radiation dose.^1,2^ Low-dose irradiation can program macrophage differentiation to an iNOS + /M1-like phenotype.^57^ In contrast, high-dose ionizing radiation can promote M2-like macrophage polarization, particularly in the context of cancer-associated fibroblasts (CAFs).^58^ Together, our results suggest that keratinocytes and macrophages engage in dynamic crosstalk in which the activation status of the infiltrating macrophage, rather than the cell number controls the fibrotic process. Keratinocytes release diverse factors, including cytokines, chemokines, and growth factors, that can attract and shape the polarization of macrophages, which is in line with a report showing a modular chemotaxis of human immune cells upon radiation of tumor cells expressing SASP chemokines.^59^ DKK3 expression by keratinocytes leads to the activation of canonical Wnt signaling and alters their secretome, polarizing them toward an M2-like/profibrotic phenotype both in vitro and in vivo (Figs. 2, 6, 7). Interestingly, DKK3 depletion in macrophages did not alter macrophage polarization toward a nonprofibrotic state, indicating that the profibrotic phenotype observed is not driven primarily by myeloid expression of DKK3 (Fig. 2, supplementary Fig. 8). The profibrotic macrophages, in turn, directly activate fibroblasts into myofibroblasts, as indicated by the upregulation of genes associated with fibrosis and basement membrane production. Their classification as myofibroblasts aligns with recent findings across multiple tissues, including skin, where DKK3 and SMA are elevated in fibroblasts under perturbed conditions.^60^ Our results also highlight the importance of fibroblast‒macrophage interactions in radiation-induced fibrosis.^49^ Indeed, our data demonstrate that radiation partially mimics the effects of DKK3 overexpression in keratinocytes (Fig. 6, supplementary Fig. 14), particularly by promoting macrophage-mediated profibrotic signaling. Irradiated wild-type keratinocytes enhanced macrophage polarization toward a CD206^high^/CD163^high^ phenotype and induced fibrosis-associated gene expression in fibroblast–macrophage cocultures. Interestingly, for both DKK3-overexpressing keratinocytes and irradiated keratinocytes, direct macrophage–fibroblast–cell contact was required for fibroblast transactivation, as activation was absent in the paracrine coculture.

Cellular senescence can contribute to radiation-induced fibrosis.^2,45,46^ We observed that DKK3 modulation in keratinocytes upregulated immune-related factors, cytokines, and chemokines, including IL-6, TGF-β, CXCL2, FGF-2, MMP,7 and IL-1β, which are associated with a SASP phenotype (Fig. 5). Thus, DKK3 may also contribute to senescence-associated skin fibrosis following radiation exposure by exacerbating the fibrotic response. The importance of profibrotic M2-like macrophages and Wnt signaling for radiation-induced skin fibrosis aligns with data from an analogous mouse model of lung fibrosis induced by 20 Gy: blocking CTGF, which acts partially by inhibiting TGF-β, attenuated fibrosis by reducing profibrotic M2-like macrophage populations and by suppressing Wnt1-inducible-signaling-pathway-protein-1 (Wisp1), while CTGF, TGF-β and Wisp1 were upregulated by radiation, and CTGF also bound to the Wnt coreceptor LRP6.^15,18^ The significance of DKK3 and canonical Wnt signaling extends to cancer, a critical consideration in radiotherapy, because potential mitigators of radiation-induced toxicity in normal tissue should not inadvertently promote tumor growth. Some studies have indicated that DKK3 expression has proapoptotic effects on tumor cells via Wnt signaling inhibition and thus has anticancer effects.^61^ In contrast, other studies have linked high DKK3 expression to protumor effects, including immunosuppression, which is correlated with poor prognosis in glioblastoma patients.^62^ Additionally, the suppression of DKK3 in pancreatic stromal stellate cells has been shown to reduce stromal density and inhibit pancreatic carcinoma progression.^63^ While our findings similarly demonstrated that suppression of DKK3 reduces canonical Wnt activity in keratinocytes and attenuates fibrosis, we identified keratinocytes as important cellular sources of DKK3 in radiation-induced skin fibrosis, with fibroblasts functioning as downstream mediators of keratinocyte-derived DKK3. The interplay among keratinocytes, DKK3, infiltrating immune cells and tissue fibroblasts thus forms a dynamic network that may help determine the severity and persistence of inflammatory skin fibrosis, highlighting the potential of DKK3 as a therapeutic target in the treatment of inflammatory skin fibrosis. Depending on the context, DKK3 can promote both fibrogenesis and the progression of certain tumors, similar to other factors, such as TGF-β, PDGF, or CTGF.^2,7,8,39^ Inhibiting these factors, which can be triggered by radiation exposure, has been shown to effectively reduce both fibrosis and cancer progression.^13–16,44,64,65^ This effect could be particularly significant for DKK3 in tumors characterized by a fibrosis-prone microenvironment, such as pancreatic carcinoma. Consequently, blocking DKK3 may not only alleviate radiation toxicity but also increase the therapeutic scope of radiotherapy for certain tumors.

In summary, we identified a central role for DKK3 in the response of the skin to radiation. ROS-induced DKK3 expression in keratinocytes orchestrates hyperproliferative and profibrotic processes following radiation-induced skin damage. Our study highlights the importance of DKK3-mediated canonical Wnt pathway activation in keratinocytes, facilitated by TGF-β signaling and profibrotic macrophage polarization, in driving radiation-induced dermatitis and fibrosis in both mice and humans. These findings position DKK3 as a promising therapeutic target for mitigating radiation-induced dermal fibrosis and potentially addressing fibrotic conditions across various anatomical sites and etiologies.

## Materials and Methods

### Study design

Mouse models of thoracic and hind limb external beam radiation damage (20 Gy) were generated in conjunction with cell-specific targeted deletion of DKK3 and promoter/reporter transgenes to identify the cell type most responsible for the phenotype observed. Specific aspects of the biology were then modeled in vitro via human cell lines and human 3D skin models via external beam radiation, reactive oxygen species, and engineered transgenes for overexpression and reporter genes. Pathway inhibitors have also been applied to identify specific pathways involved.

### Mice

Female 12-week-old mice on a C57BL6/N background were used for the experiments. The mice were kept under specific pathogen-free conditions in the animal facility of the German Cancer Research Center (DKFZ). All animal experiments were approved by the DKFZ institutional review board (IRB) and governmental authorities (Regierungspräsidium, Karlsruhe, Germany). The relevant animal care numbers were G-270/15, G-280/19, G-255/20 and G-113/21.

### Generation of DKK3 knockout mice

An embryonic stem cell clone containing a floxed allele of DKK3 (clone EPD0642_3_H05) was purchased from The Knockout Mouse Project (KOMP). The floxed DKK3 line was bred with Cre-deleter mice to generate global DKK3 knockout mice or with Cre recombinase-expressing animals under the control of the Keratin14 (K14) promoter^36^ to generate keratinocyte-specific knockouts. Cell-specific knockouts of the DKK3 gene in macrophages (leukocytes) and fibroblasts were generated by breeding Colony Stimulating Factor 1 Receptor (CSF1R) promoter^37^ or inducible Platelet-Derived Growth Factor Receptor (PDGFR) promoter Cre-recombinase mice,^38^ respectively. All the DKK3 mouse lines have been bred for many years in our mouse facility. No deleterious phenotype was observed at any time (i.e., no behavioral changes or reproductive abnormalities).

### Generation of DKK3/Wnt dual-reporter mice

The mouse strains Tg (DKK3-luc/mCherry) #Hjg^30^ and Tg (TCF/Lef1-HIST1H2BB/EGFP) 61Hadj^41^ were crossed on a C57BL6/N strain background to generate a DKK3-LCh x TCF/LEF dual reporter mouse line with luciferase and mCherry expression under the control of the DKK3 promoter and eGFP expression under the control of the β-catenin-responsive TCF promoter.

### Mouse irradiation

Mouse experiments were carried out at the animal facility and the division of Molecular and Radiation Oncology at DKFZ in Heidelberg. The mice were weighed and anesthetized with 70 mg/kg ketamine and 7 mg/kg xylazine. Five to ten minutes after anesthesia, the mice were fixed in a radiation device designed for the thorax (described in refs. ^12,15^) or hind limb radiation. The thoracic field size was approximately 2 cm (transversal) × 1 cm (craniocaudal) to partially shield the lungs and other body parts. The hind limb field size was 1 cm². The mice were irradiated via a MultiRad 225 X-ray system with a dose rate of 5.53 Gy/min, a tube voltage of 200 kV, and a tube current of 17.8 mA at shelf 6. A 0.5 mm copper filter was used to increase the hardness of the photon beam. The mice were scored according to supplementary Table 6.

### Histology and immunohistochemistry

Immediately after the mice were sacrificed, their skin tissue was collected and fixed in 4% formalin for one week. Paraffin-embedded skin sections (4 μm) were cut, and histological (H&E, Goldner) and a panel of IHC stainings (see antibody details in supplementary Table 3) were performed via standard protocols. Five images of IHC and Goldner staining at random positions were acquired from each section/stained sample via a Leica LEITZ DMRD microscope at 100x magnification. Quantification of the IHC and Goldner staining results was semiautomatically performed via ImageJ software via custom algorithms. Epidermal thickness was measured manually at approximately 15 positions in each H&E section and averaged for each mouse. The percentage of marker-positive cells was defined on the basis of the positive area/selected area (dermis was manually selected) and averaged from all 5 images/mouse.

### In situ hybridization

Immediately after the mice were sacrificed, skin tissue was collected, fixed in 4% buffered formaldehyde for 24 h and paraffin-embedded. Human skin samples were obtained via paraffin embedding. Paraffin-embedded skin sections (4 μm) were cut for DKK3 in situ hybridization (ISH) with an RNAscope™ 2.5 High Definition (HD)-RED Assay (ACD, 322350) and a DKK3 RNAscope probe (ACD, 400931 (Mm) and 415781 (Hs)). ISH was performed following the manufacturer’s instructions. ISH was quantified in 5 HPFs (high-power fields, 40×) per sample by counting the number of spots in basal keratinocytes.

### Confocal imaging of DKK3/Wnt dual-reporter mouse skin

Immediately after sacrifice, mouse skin samples were removed and fixed in 4% buffered formaldehyde for 24 h. The samples were then incubated successively in 15% sucrose and 30% sucrose, frozen in Tissue-Tek® O.C.T.™ Compound (Sakura Finetek) on dry ice and stored at -80 °C until analysis. Four-micron-thick skin sections were cut via a microtome and stained with DAPI. Fluorescence images were acquired via a Leica TCS SP5 confocal microscope (Leica Biosystems) with a 63x/1.4 oil immersion objective lens and excitation at 405 nm (DAPI), 488 nm (DKK3), and 561 nm (Wnt signaling).

### Skin cell isolation

Fresh mouse skin was washed in cold PBS and then predigested in 2 ml of PBS containing 1 mg/ml Dispase II + 50 U/ml DNAse I for 45 minutes at 37 °C. After the reaction was stopped by the addition of 1 ml of FCS, the samples were immersed in 300 µl of DMEM with 10% FCS, 800 U/ml collagenase IV + 50 U/ml DNase I (digestion buffer 2) and cut into small pieces. A total of 1.5 ml of digestion buffer 2 was added, and the samples were further digested in a thermoshaker at 900 rpm and 37 °C for 1 h. Four microliters of 0.5 M EDTA were added to stop digestion, and after 10 min of incubation, the samples were passed through a 70 µm cell strainer and washed with 20 ml of HB buffer (Hanks balanced salt solution + 2 mM EDTA + 0.5% BSA). The cells were pelleted by centrifugation at 350× g and 4 °C for 7 minutes, the supernatant was removed, and the cells were resuspended in 200 µl of FCM buffer (PBS/2% FCS/2 mM EDTA/0.1% NaN3) immediately before FCM staining.

### Flow cytometry (FCM)

Surface-antigen staining was performed according to standard protocols (see antibody details in supplementary Table 4). The background was blocked with an anti-CD16/CD32 antibody (101302, Biolegend), and dead cells were stained with Zombie Aqua (423105, Biolegend). Intracellular staining was performed after fixation and permeabilization of the cells via the FIX & PERM Kit (GAS003, Thermo Fisher). Samples were acquired with a LSR Fortessa cytometer (BD) and analyzed with FlowJo 10.9 software (see the gating strategy in supplementary Fig. 15).

### Cell culture

Human immortalized N/TERT-1 keratinocytes were kindly provided by Prof. Dr. Birgit Schittek, University of Tübingen. N/TERT-1- and N/TERT-1-derived cell lines were maintained in K-sfm media supplemented with 1% penicillin, human recombinant epidermal growth factor (hrEGF) and bovine pituitary extract (BPE) at 37 °C and 5% CO_2_. Human immortalized K4 fibroblasts were cultured in Dulbecco’s modified Eagle’s medium (DMEM) supplemented with 10% FCS and 1% penicillin at 37 °C and 5% CO_2_.

### Reporter cell lines

An in-house vector platform was used for the generation of interacting stable transgenes in keratinocyte and fibroblast lines.^42^ The system uses dual plasmid vectors, one for monitoring pathway activity and providing constitutive expression of the Tet repressor protein and a second vector for Tet-controlled overexpression of target genes. These vectors contain Sleeping Beauty (SB) transposase inverted repeats for efficient incorporation of multiple transgenes into host cell DNA. To this end, the transfection reaction included pCMV(CAT)T7-SB100 (Addgene, #34879), which encodes the SB transposase to drive integration. Two or three plasmids (depending on the experiment) were cotransfected via an NEON electroporation device (using 1150 V, 30 ms, 1 pulse in 100 μl NEON-tips) and selected as described previously.^42^ The reporter vectors use a secreted version of *Gaussia* luciferase as a reporter gene, allowing the monitoring of reporter activity in the cellular growth medium under the control of the β-catenin-responsive TCF/LEF promoter pSBTR (Addgene, #79482) or the SMAD-responsive promotor pSBTR (Addgene, #79483). The β-catenin-responsive TCF/ LEF promoter pSBTR was used alone or in combination with pcDNA6.2ITR-Zeo-CMV/TO-DKK3 for simultaneous induction of DKK3 overexpression in N/TERT-1 cells (supplementary Figs. 16, 17, 18). The SMAD-responsive promoter pSBTR-plasmid was used to generate K4 TGF-β–SMAD reporter cells (supplementary Figs. 16, 18).

### Measurement of secreted *Gaussia* luciferase activity

A *Gaussia* luciferase assay kit (GeneCopoeia, MD, USA) was used for quantification of canonical Wnt (β-catenin) activity in N/TERT-1 keratinocytes and TGF-β–SMAD pathway activity in K4 fibroblasts with the respective reporters. A total of 10 µl of cell culture supernatant was added to 100 µl of preprepared substrate/buffer solution, incubated for 30 seconds, and measured via a Berthold Lumat LB 9507 luminometer.

### DKK3 modulation in keratinocytes

N/TERT-1 keratinocytes with Dox-inducible DKK3 overexpression were starved for 24 h in supplement-free K-sfm medium and then stimulated with 0.2 µg/ml doxycycline. DKK3 was knocked down in N/TERT-1 cells with 20 nM DKK3 siRNA (GeneGlobe ID SI00100933) via Hyperfect transfection reagent, which included nontargeting siRNA, and mock transfection, which served as a control (all reagents from Qiagen, Hilden, Germany). DKK3 modulation was validated by ELISA, RT-qPCR, and Western blotting (see supplementary Fig. 6).

### TGF-β1 modulation in keratinocytes

N/TERT-1 keratinocytes with Dox-inducible DKK3 overexpression and the canonical Wnt reporter were cultured under 2D or 3D skin model conditions. Twenty-four hours after Dox-induced DKK3 overexpression, the cells were treated with 50 ng/ml recombinant TGF-β1 protein (rTGF-β1; Peprotech, 100–21C), 5 µM TGF-β receptor 1 and 2 kinase inhibitor LY2109761 (Sigma Aldrich, SML2051) or 2.5 µg/ml TGF-β-blocking antibody (Invitrogen, MA5--23795) and assessed for canonical Wnt activity after 24 h or epidermis thickness and Ki67 expression in the 3D model after 72 h.

### Keratinocyte-conditioned macrophage (KcMф) preparation

Conditioned media were collected from N/TERT-1 keratinocytes 7 days after DKK3 modulation (see above). Whole blood was drawn from a healthy donor (approved by the LMU Clinic Ethics Commission, Project No. 19-159), and peripheral blood mononuclear cells (PBMCs) were extracted via centrifugation via Biocoll separation solution (Biosell, L6115). Monocytes were isolated from PBMCs via CD14 beads and a MACS separator (Miltenyi Biotech, Köln, Germany) and matured into macrophages in AIM V medium (Thermo Fisher) supplemented with 2% human serum and 25% keratinocyte-conditioned medium for 7 days.

### Macrophage/fibroblast coculture

Preseeded K4 fibroblasts with a TGF-β–SMAD reporter were cocultured with KcMф at a 1:1 ratio or incubated with DMEM containing 25% medium from KcMф. Twenty-four hours after direct/indirect coculture, the medium was collected for Gaussia luciferase-based quantification of TGF-β–SMAD pathway activity, and the expression of fibrosis-related genes was quantified in the cells via RT‒qPCR.

### 3D skin model

A human epidermal equivalent 3D model^43^ was created using N/TERT-1 keratinocytes with Dox-inducible DKK3 overexpression and a canonical Wnt reporter. Three days after stimulation with 0.2 µg/ml Dox, the medium was collected for quantification of DKK3 expression and canonical Wnt activity (secretion of *Gaussia* luciferase). The 3D skin constructs were fixed in 4% paraformaldehyde and paraffin-embedded. Two-micron-thick sections were cut for H&E staining and Ki67 immunohistochemistry (1:100 dilution, Abcam, ab1667).

### Cell irradiation

The cells were irradiated via a Multirad 225 X-ray system with a dose rate of 2.15 Gy/min, a tube voltage of 200 kV, and a tube current of 17.8 mA. A 0.5 mm copper filter was used to increase the hardness of the photon beam.

### ROS measurement

Endogenous ROS levels were determined via the 2’,7’-dichlorodihydrofluorescein diacetate (H2DCFDA) assay (Thermo Fisher, C6827). A total of 10000 N/TERT-1 keratinocytes with a canonical Wnt reporter were seeded per well in 96-well black clear bottom plates (Nunc, 165305). The cells were irradiated with 4 Gy and incubated for 2 or 24 h, or treated with different concentrations of rotenone (Sigma Aldrich, R8875) for 12 h and then gently washed with 1x PBS. One hundred microliters of 10 µM H2DCFDA (1 mM DMSO stock) in 1x PBS was added to each well, and the mixture was incubated at 37 °C for 30 minutes. The fluorescence (ex/em 545/600 nm) was recorded with a microplate reader (CLARIOstar, BMG).

### Clonogenic survival

N/TERT-1 cells were seeded in 25 cm^2^ flasks in triplicate with increasing cell numbers/dose group and irradiated 24 h later with 0, 1, 2, 4, 6, or 10 Gy. The samples were incubated at 37 °C and 5% CO_2_ until colony formation occurred, after which they were stained with crystal violet. Colonies with >50 cells were counted via a light microscope, and the plating efficiency (PE) and surviving fraction (SF) were calculated according to the following formulas:

$${PE}=\frac{{number\; of\; colonies\; formed}}{{number\; of\; cells\; seeded}}$$

$${SF}=\frac{{PE}({irradiated\; group})}{{PE}({unirradiated\; controls})}$$

SF was fitted with SigmaPlot 14 software according to the linear‒quadratic model.

### Proliferation assay

N/TERT-1 cells (5 × 10^4^) were seeded into 25 cm^2^ flasks in triplicate/group and irradiated with 0, 1, 2, 4, 6, or 10 Gy after 24 h. Three days after irradiation, the number of living cells in each flask was counted via a Neubauer chamber after Trypan blue staining.

### Measurement of DNA double-strand breaks, the cell cycle, and apoptosis

Flow cytometric measurement of the DNA damage response was performed as previously described.^66^ In brief, the cells were fixed with 3% paraformaldehyde, permeabilized with ice-cold 70% ethanol, and stained with antibodies against γH2AX (Alexa Fluor 488, Biolegend, San Diego, CA, USA) and active caspase-3 (Alexa Fluor 647, BD Biosciences, Heidelberg, Germany). The data were acquired via an LSRII flow cytometer and analyzed via FlowJo 10.7.1 software. In addition, stained cells were air-dried on glass coverslips and embedded with Fluoromount G (Southern Biotech) on glass slides for microscopic evaluation of γH2AX foci via a semiautomatic system (MetaSystems, Altlußheim, Germany).

### Senescence assay

Senescence-associated β-galactosidase staining was performed as previously described.^47,59^ In brief, DKK3-modulated keratinocytes were irradiated and seeded into individual wells containing sterile coverslips. Four days later, the cells were fixed and incubated at 37 °C in staining solution containing X-gal at pH 6.0 (Cell Signaling Technology, 9860S), washed, and incubated with 1 µg/ml DAPI. Coverslips were mounted on microscope slides, and 5 random fields/samples were acquired via brightfield and fluorescence microscopy. Perinuclear SA-β-Gal staining was quantified semiautomatically via ImageJ v.1.52 d. Three independent experiments with 3 triplicates were performed.

### ELISA

A human DuoSet ELISA (R&D Systems) kit was used for DKK3 protein measurement in the cell culture supernatants according to the manufacturer’s instructions. The results were recorded via a SpectraMax M5e Microplate Reader (Molecular Devices, Biberach a der Riss, Germany) at 562 nm absorbance.

### Multiplex antibody-based protein profiling

A custom panel of chemokines and growth factors, including human TGF-β1, IL-4, IL-6, IL-10, IL-1β, CXCL8, CXCL10, IFN-γ, FGF, GM-CSF, M-CSF, PDGF-BB, and TGF-α, was measured in cell culture supernatants via the LEGENDplex assay platform (BioLegend, San Diego, USA). Data were acquired via an Canto I flow cytometer and analyzed with LEGENDplex Data Analysis Software.

### Western blot

Western blotting was performed using a rabbit anti-human DKK3 antibody (Abcam) and an anti-human TGF-β1 antibody (for latent TGF-β1, Thermo Fisher). The rabbit endogenous anti-human β-actin antibody (Cell Signaling Technology) was used as an internal protein control. Protein bands were quantified via Fiji (ImageJ) software.

### RT‒qPCR

RNA was isolated from cells via the PureLink™ RNA Mini Kit (Invitrogen, 12183018 A) and reverse-transcribed into cDNA via the SuperScript II Reverse Transcriptase Kit (Invitrogen, 10328062). Gene expression was measured via a SYBR Green real-time PCR kit (Sigma‒Aldrich, QR0100-1KT) via a Light Cycler 480 (Roche) and quantified relative to 18S rRNA via the ΔΔCt method. The primer sequences are listed in supplementary Table 5. Enzyme-free RT‒qPCR and sample-free qPCR were included, and melting curve analysis was performed to control for unspecific amplification.

### nCounter gene expression analysis

The expression levels of 770 fibrosis-related genes were measured with the nCounter SPRINT Profiler via the human fibrosis panel V2 assay (NanoString Technologies, Munich, Germany). N/TERT-1-Wnt-DKK3 cells were stimulated with 0.2 µg/ml doxycycline to induce DKK3 expression. DKK3 RNA knockdown in N/TERT-1 cells was achieved via transfection via Hyperfect transfection reagent (Qiagen, Hilden, Germany) with 20 nM DKK3 siRNA (GeneGlobe ID SI00100933) or nontargeting siRNA as a control (NT scramble). RNA was isolated 24 hours after treatment. In brief, 50 ng of RNA was mixed with the assay-specific Reporter CodeSet and Capture ProbeSet in the provided hybridization buffer and incubated at 65 °C overnight. The hybridized samples were then loaded into an nCounter cartridge and immediately measured. QC and data analysis were performed with ROSALIND® (https://rosalind.onramp.bio/). Counts were normalized lanewise to the geometric mean of the normalizer probes. The NormqPCR R library1 was used to select normalizer probes via the geNorm algorithm. Fold changes and *p*-values were computed according to Nanostring’s guidelines. Clustering of genes for the final heatmap of differentially expressed genes was performed via the partitioning around medoids (PAM) method.

### Human tissue samples

Human skin samples were obtained from routine biopsies. The ethics committees of the University of Münster and University of Marburg approved the use of human skin biopsies for this project under file numbers 2021–510-f-S and 169/19, respectively. The ethics committee of Peking University Third Hospital Medical Science Research Ethics Committee approved the use of human skin biopsies for this project under file numbers 2025–527–02. According to the scientific law of the city of Hamburg, human skin samples could be used for scientific research and thus for this project without further approval. The patient samples included 5 normal skin samples (5 male, 0 female; ages 58–82); 3 keloid scar samples (3 male, 0 female; ages 45–82); 12 radiodermatitis samples (6 male, 6 female; ages 37–84); 7 Lichen sclerosus et atrophicus (LSA) samples (1 male, 6 female; ages 36–79); and 4 morphoea samples (1 male, 3 female; ages 45–69).

### Single-cell RNA sequencing analysis

Single-cell RNA sequencing data from human skin (GSE193807; 8291 radiation-exposed and 8534 healthy control cells).^50^ were processed via Seurat (v5.0.1). The cells subjected to quality control were retained if they expressed 200–6000 genes, 1000–50,000 UMIs, ≤15% mitochondrial genes, and ≤100% ribosomal genes, yielding 6336 irradiated and 5455 control cells. No significant batch effects or cell cycle variations were detected; thus, no corrections were applied. The data were normalized (scale factor = 10,000), and 2000 highly variable genes were selected. Principal component analysis (PCA) was performed and the first 11 principal components were used (determined by elbow plot), followed by UMAP dimensionality reduction (dims 1–20) and clustering at a resolution of 0.1. Cell types were annotated via top expressed gene ranking analysis via cell clustering and CellMarker 2.0 validation via canonical markers (e.g., CLDN1/FERMT1 for epidermal keratinocytes [eKC]; SPRR2A/KRT6A for differentiated keratinocytes [dKC]), which identified 11 skin-specific populations: eKCs, dKCs, fibroblasts (FBs), vascular endothelial cells (vECs), sweat gland ductal cells (SGCs), vascular smooth muscle cells (vSMCs), Schwann cells (SCs), CD8-positive T lymphocytes (CD8+TCs), macrophages (Møs), and mast cells (MCs). Subsequent analysis focused on DKK3 expression in the eKC and dKC subpopulations to assess radiation-induced changes. Analysis of the dataset (8,291 radiation-exposed cells vs. 8,534 nonirradiated cells) revealed a significant increase in global DKK3 expression when all cells were analyzed collectively (*P* < 0.001), indicating a systemic response to radiation. Concurrently, keratinocyte subpopulations exhibited divergent shifts: basal epidermal keratinocytes (eKCs; undifferentiated, proliferative state) increased dramatically postirradiation (505 vs. 7 cells in controls), whereas terminally differentiated epidermal granular keratinocytes (dKCs; barrier-forming state) declined significantly (284 vs. 197 cells). Pooled keratinocyte (eKC + dKC) analysis confirmed that DKK3 was elevated (*P* < 0.001), with eKC dominating the postirradiation keratinocyte compartment. By comparing the irradiated group (IR) and the control group (Con), we identified DKK3 and related genes that were significantly differentially expressed. To categorize cells on the basis of DKK3 expression levels, we employed an optimized cutoff approach owing to the abundance of zero values typical of single-cell gene expression data.

We systematically tested multiple quantiles from 10% to 90% at 5% intervals to determine the optimal cutoff value that maximizes group differences. Using Wilcoxon rank-sum tests, we identified the best-performing quantile at 55%, corresponding to a cutoff value of 0.746. This optimized threshold was then applied to stratify the cells into DKK3^high^ and DKK3^low^-expression groups.

### Statistics

The quantification of the histology and immunohistology data was performed with Fiji (ImageJ) software (National Institutes of Health). FCM data were analyzed with FlowJo 10.7.1/10.9. software (BD). All the graphs and statistical analyses were generated via Microsoft Excel or GraphPad Prism 8.0 software. Unless otherwise indicated, the data are presented as the mean ± standard error of the mean (SEM). For parametric distributions, comparisons between two groups were performed via unpaired or paired two-tailed Student’s t tests, and comparisons between more than two groups were performed via one-way or two-way ANOVA with Tukey’s multiple comparisons tests. A *p*-value of <0.05 was considered statistically significant. A linear‒quadratic model was used to fit the surviving fraction in the colony formation assays (SigmaPlot 14).

## Data and materials availability

All the data and materials used in the analysis are available in some form to any researcher for purposes of reproducing or extending the analysis. Materials transfer agreements (MTAs) are required from the German Cancer Research Center (DKFZ), Heidelberg, for access to the mouse lines. The nCounter gene expression data (NanoString) are available at the European Bioinformatics Institute (EMBL): https://www.ebi.ac.uk/biostudies/arrayexpress/studies/E-MTAB-16037?key=51a4f0aa-9287-412b-b898-2733993107b1 (10.6019/E-MTAB-16037). The R code used to analyze the single-cell sequencing data is available in the public database zenodo.org: 10.5281/zenodo.17583288.

## Supplementary information

Supplementary Material

Supplementary Data 1_uncropped WB
