# Supplementary material for: Wnt-associated DKK3 in keratinocytes mediates radiation-induced hyperplasia, dermatitis and skin fibrosis
Source: Signal Transduct Target Ther. 2026 Feb 2;11:41. doi: 10.1038/s41392-025-02541-z (PMC12864833; doi:10.1038/s41392-025-02541-z)
Supplement: Supplementary file 1 — Supplementary Material [file 41392_2025_2541_MOESM1_ESM.docx]

Supplementary Materials for

Wnt-associated DKK3 in keratinocytes mediates radiation-induced hyperplasia, dermatitis and skin fibrosis

Li Li, Ramon Lopez Perez, Khuram Shehzad, Richard Jennemann, Claudia Schmidt, Thomas Walle, Alexandra Tietz-Dahlfuß, Elisabeth Grimm, Joscha A. Kraske, Peter Häring, Uladzimir Barayeu, Tobias P. Dick, Luxi Ye, Stephan A. Braun, Michael Hertl, Thomas Worzfeld, Thorsten Wiech, Huihui Ji, Jing Su, Jonathan M. Schneeweiss, Muzi Liu, Katharina Kommoss, Matthias Heikenwälder, Bingwen Zou, Sabrina Mücklich, Kerstin Steinbrink, Verena K. Raker, Wenjun Wu, Elfriede Noessner, Hermann-Josef Gröne, Peter J. Nelson^†^, Roger Sandhoff^†^, Peter E. Huber^†^

Correspondence to: peter.nelson@med.uni-muenchen.de, r.sandhoff@dkfz.de or p.huber@dkfz.de

**This file includes:**

Supplementary Materials and Methods

Supplementary Figures 1 to 21

Supplementary Tables 1 to 6

Supplementary References

**Other Supplementary Materials for this manuscript include the following:**

Supplementary Data 1: Uncropped Western blot images (separate file)

Supplementary Materials and Methods

Resazurin viability assay

4000 cells were seeded into the wells of a 96-well plate and after 24 h DKK3 was modulated as described in the methods section of the article. At 24, 48 and 72 h after DKK3 modulation, resazurin (20 mg/mL stock solution in DPBS) was added to a final concentration of 25 µg/mL into the medium of the cells or medium only controls and incubated for 1.5 h at 37°C, 5% CO_2_ atmosphere. Fluorescence at 530 nm excitation and 590 nm emission wavelength was then measured with a plate reader. Fluorescence of medium only controls was subtracted and sample values were normalized to the mean of control or NT siRNA groups, respectively.

Supplementary Figure 1.


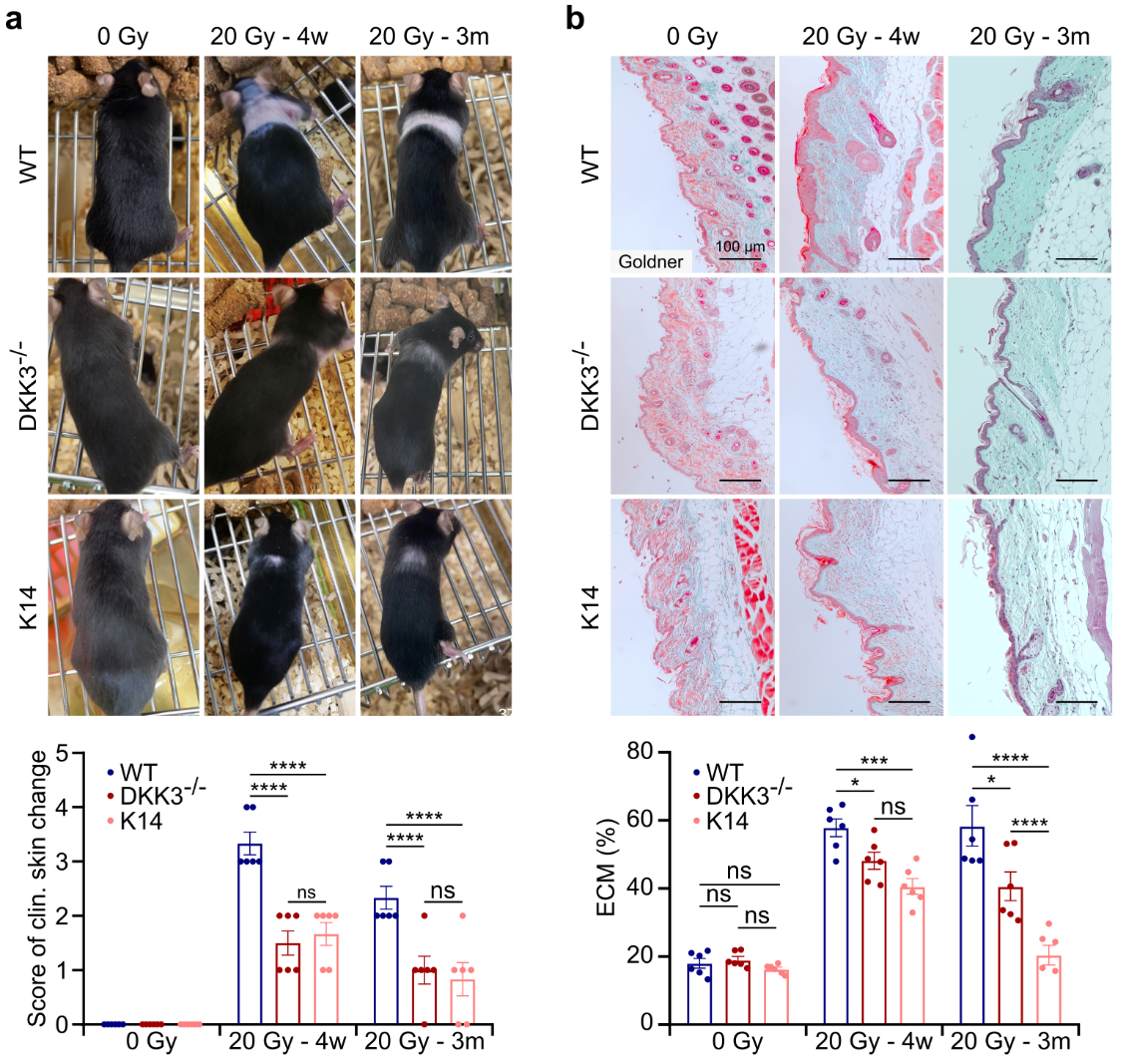


**Supplementary Figure 1**. **Time course of radiation-induced changes in mouse skin.** For direct comparison of radiation effects at different time points in one figure, this supplementary figure 1 contains mouse pictures and histological sections also presented in figures 1, 2 and supplementary figure 6. The thorax of wildtype (WT), global DKK3 knockout (DKK3^-/-^) or keratinocyte-specific DKK3 knockout (K14) mice (*n* = 6/group) was partially irradiated with 20 Gy. Injury was scored at 4 weeks and 3 months after irradiation based on criteria established by the Radiation Therapy Oncology Groups (RTOG) and the European Organization for Research and Treatment of Cancer (EORTC), where 0 represents no radiation effects seen and 5 represents effects leading to death (detailed criteria in Table S5).^1^ Skin sections of the irradiated versus the non-irradiated (0 Gy) thoracic region at 4 weeks and 3 months were stained with Goldner staining to determine collagen deposition (percentage of extracellular matrix, ECM %). (**a**) Representative mice images (top panels) and clinical injury scores (bottom panels). (**b**) Representative images (top panels) and quantifications (bottom panels) of ECM % in skin sections with Goldner staining. Scale bars: 100 μm. The data are presented as the means ± SEMs. Statistical analysis was performed via one-way ANOVA with Tukey’s multiple comparisons test, *P<0.05, **P<0.01, ***P<0.001, ****P<0.0001.

Supplementary Figure 2.


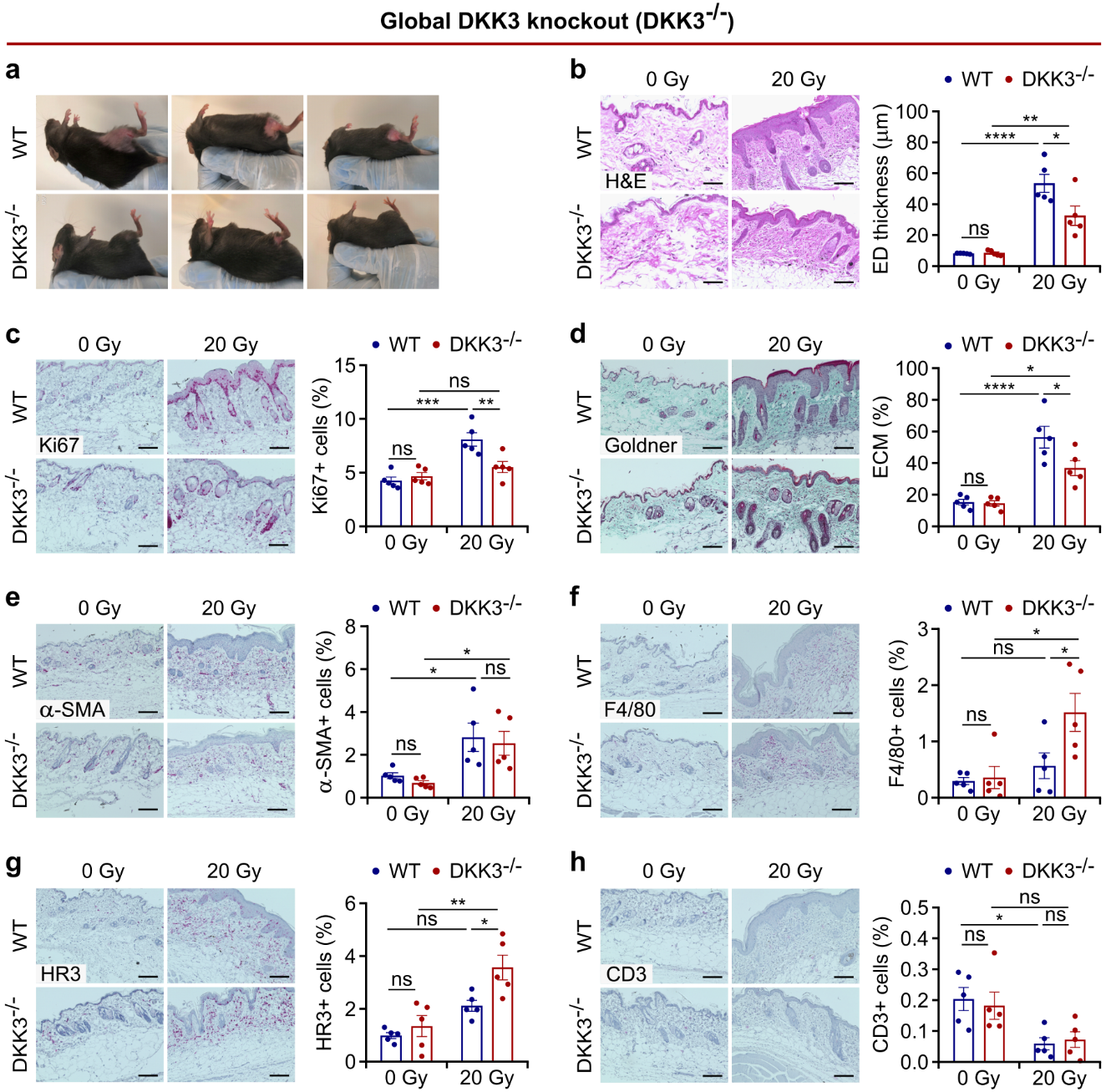


**Supplementary Figure 2.** **Global DKK3 knockout mitigates dermatitis 4 weeks after irradia-tion in the hind limb mouse model.** Wildtype (WT) and global DKK3 knockout (DKK3^-/-^) mice (*n* = 5/group) were analyzed 4 weeks after 20 Gy irradiation of one hind limb versus the unirradiated contralateral hind limb (0 Gy). (**a**) DKK3^-/-^ mice were protected from radiation-induced alopecia. (**b-h**) Representative images (left panels) and quantifications (right panels) of skin sections stained for the indicated markers. ED: epidermis. Scale bars: 50 μm. This figure is adapted from the doctoral dissertation of the first author.^2^ The data are presented as the means ± SEMs. Statistical analysis was performed via two-way ANOVA with Tukey's multiple comparisons test, *P<0.05, **P<0.01, ***P<0.001, ****P<0.0001.

Supplementary Figure 3.

**
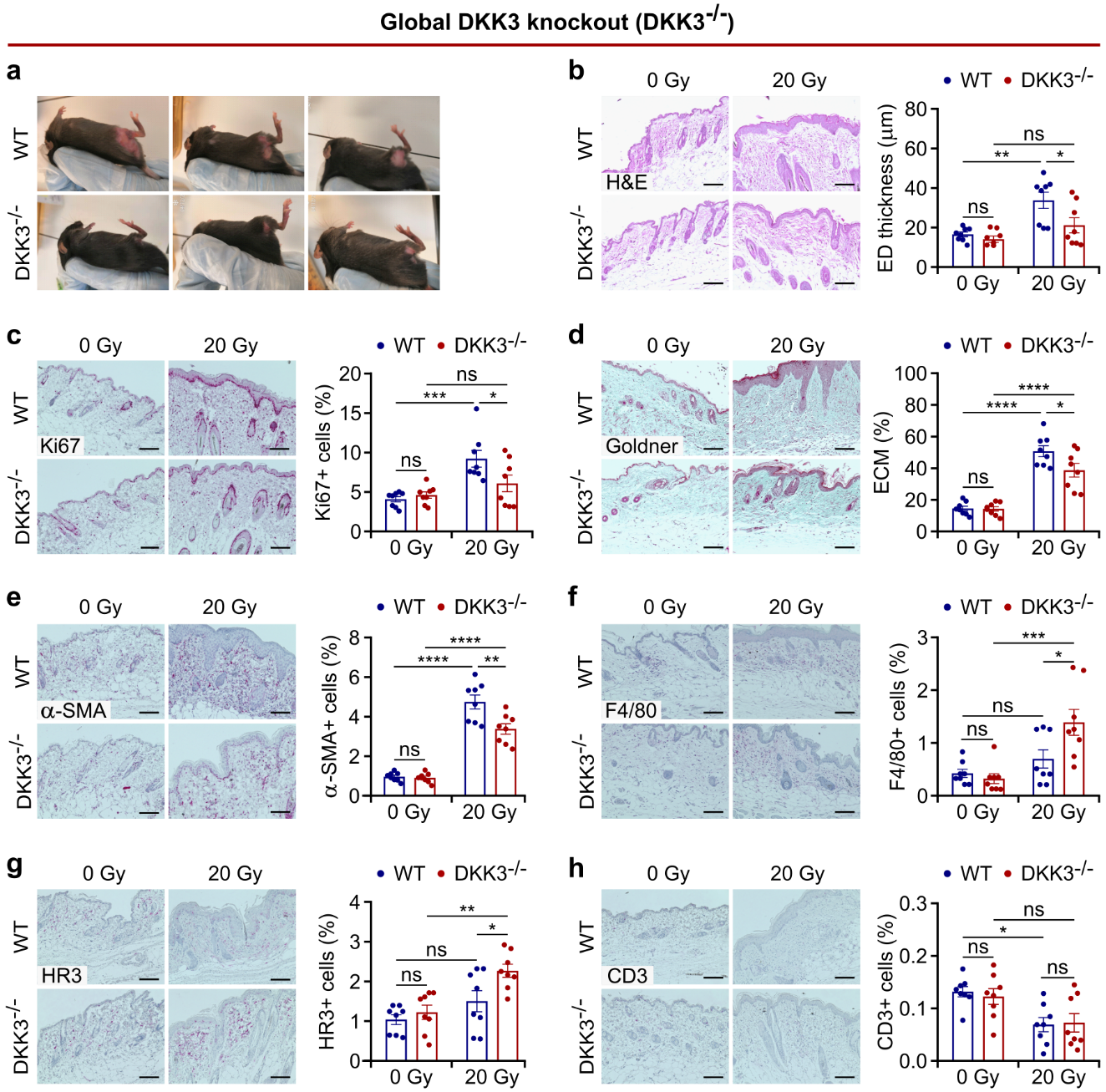
**

**Supplementary Figure 3. Global DKK3 knockout mitigates dermatitis 8 weeks after irradia-tion in the hind limb mouse model.** Wildtype (WT) and global DKK3 knockout (DKK3^-/-^) mice (*n* = 8/group) were analyzed 8 weeks after 20 Gy irradiation of one hind limb versus the unirradiated contralateral hind limb (0 Gy). (**a**) DKK3^-/-^ mice were protected from radiation-induced alopecia. (**b-h**) Representative images (left panels) and quantifications (right panels) of skin sections stained for the indicated markers. ED: epidermis. Scale bars: 50 μm. This figure is adapted from the doctoral dissertation of the first author.^2^ The data are presented as the means ± SEMs. Statistical analysis was performed via two-way ANOVA with Tukey's multiple comparisons test, *P<0.05, **P<0.01, ***P<0.001, ****P<0.0001.

Supplementary Figure 4.

**
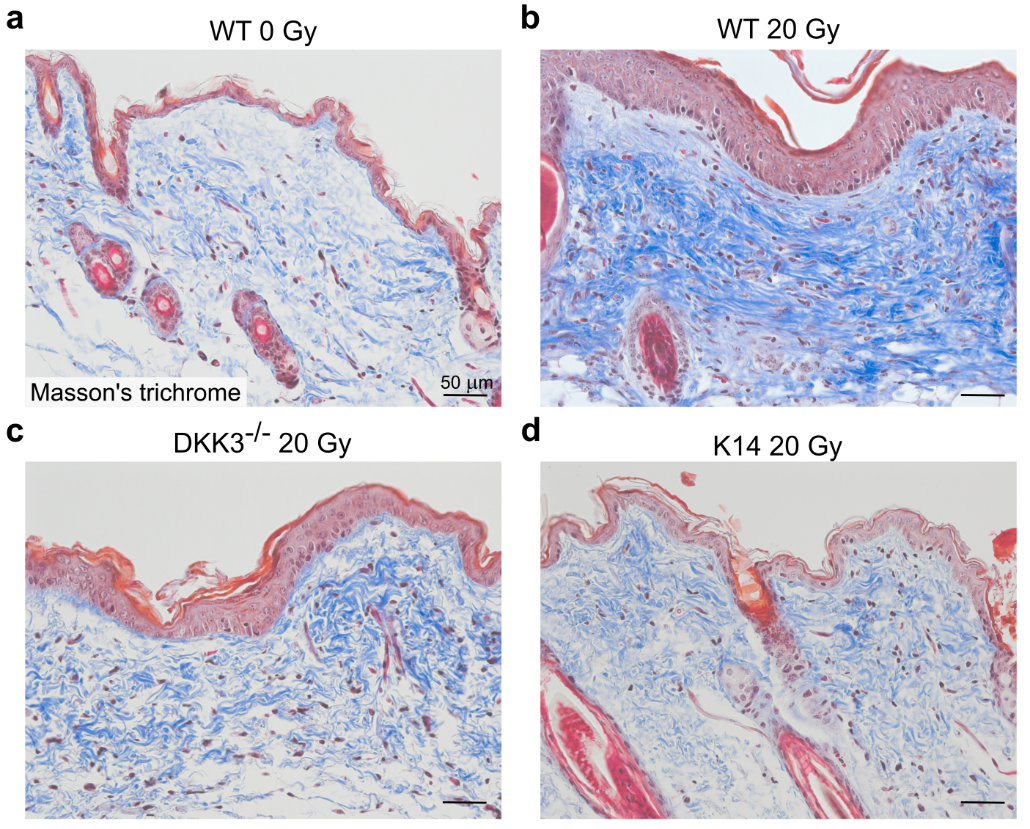
**

**Supplementary Figure 4.** **Masson’s trichrome staining of skin from global and keratinocyte-specific DKK3 knockout mice at four weeks after thoracic irradiation.** (**a**) Non-irradiated wildtype (WT) mouse skin was compared with 20 Gy irradiated skin from (**b**) WT, (**c**) global DKK3 knockout (DKK3^-/-^) and (**d**) keratinocyte-specific knockout (K14) mice at four weeks after treatment. Radiation-induced signs of fibrosis in WT mice were reduced in DKK3^-/-^ and K14 mice. Scale bars: 50 μm.

Supplementary Figure 5.

**
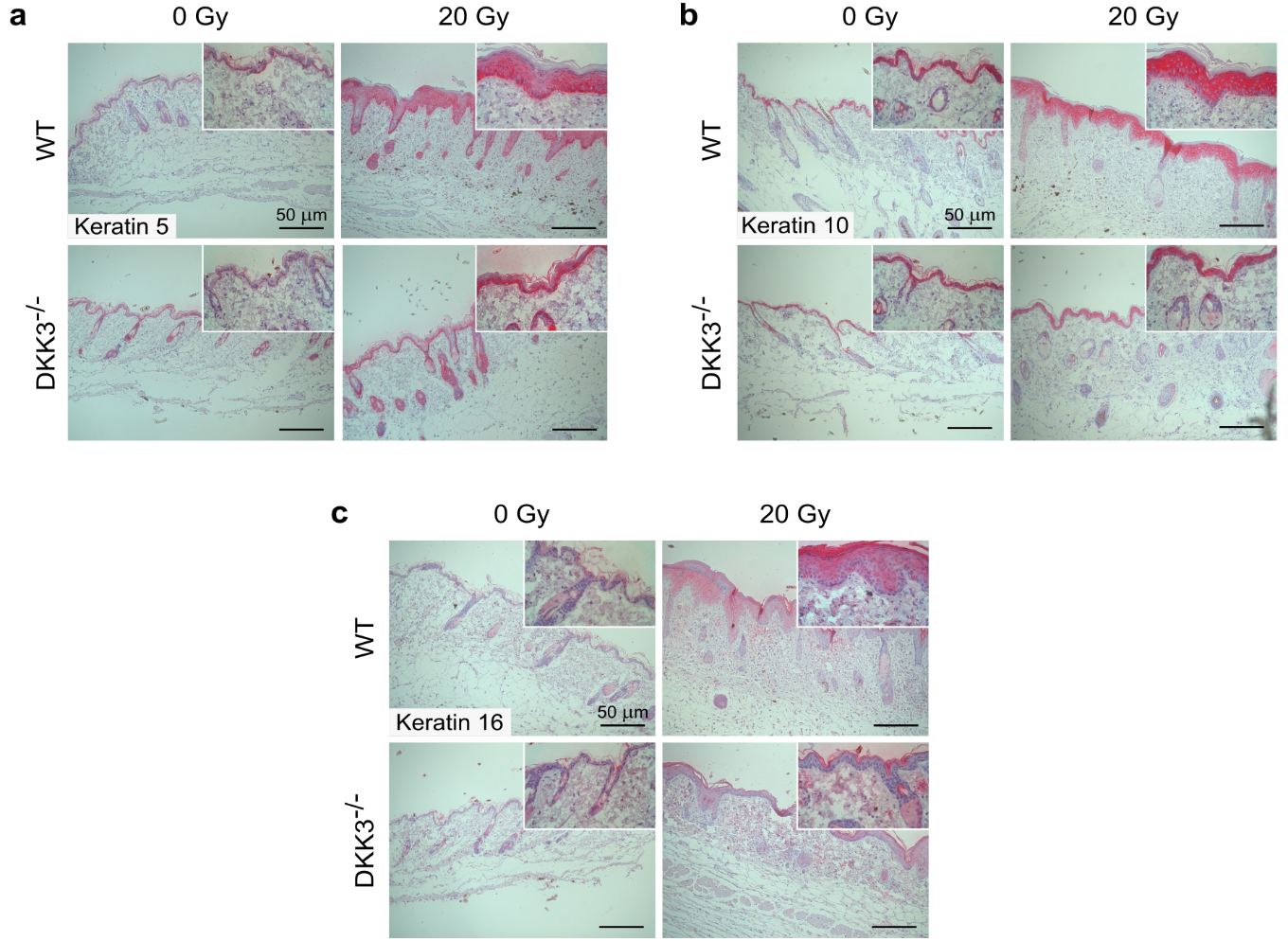
**

**Supplementary Figure 5. Radiation-induced DKK3 drives hyperproliferation of keratino-cytes.** One hind limb of wildtype (WT) and global DKK3 knockout (DKK3^-/-^) mice was irradiated with 20 Gy. Skin sections of the irradiated hind limb versus the non-irradiated (0 Gy) second hind limb were immunohistochemically stained for (**a**) keratin 5, (**b**) keratin 10 and (**c**) keratin 16 at 8 weeks after irradiation. Pronounced signs of (**a,b**) radiation-induced hyperplasia and (**c**) stressed keratinocytes were apparent in the epidermis of WT mice, which were attenuated in the DKK3^-/-^ mice. Scale bars: 50 μm.

Supplementary Figure 6.

**
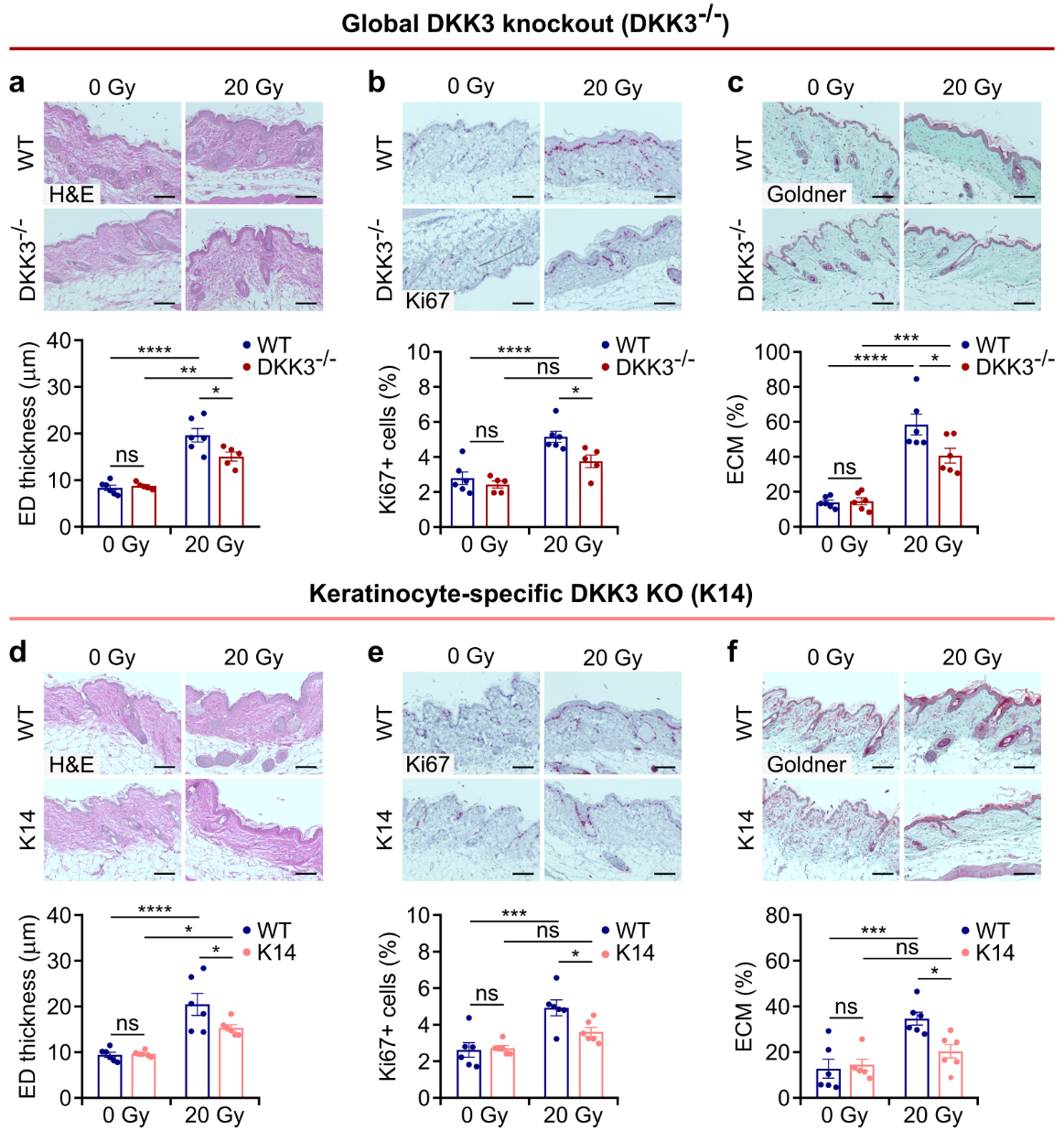
**

**Supplementary Figure 6.** **Global and keratinocyte-specific DKK3 knockout protects mice from radiation-induced hyperplasia and fibrosis at 12 weeks after irradiation in the thoracic mouse model.** (**a-c**) Global DKK3 knockout (DKK3^-/-^) mice and (**d-f**) keratinocyte-specific DKK3 knockout (K14) mice were analyzed 12 weeks after 20 Gy thoracic irradiation (*n* = 6/group). Representative images (top panels) and quantifications (bottom panels) of skin sections stained for (**a,d**) H&E, (**b,e**) Ki67 and (**c,f**) Goldner are shown. ED: epidermis. Scale bars: 50 μm. The data are presented as the means ± SEMs. Statistical analysis was performed via two-way ANOVA with Tukey's multiple comparisons test, *P<0.05, **P<0.01, ***P<0.001.

Supplementary Figure 7.

**
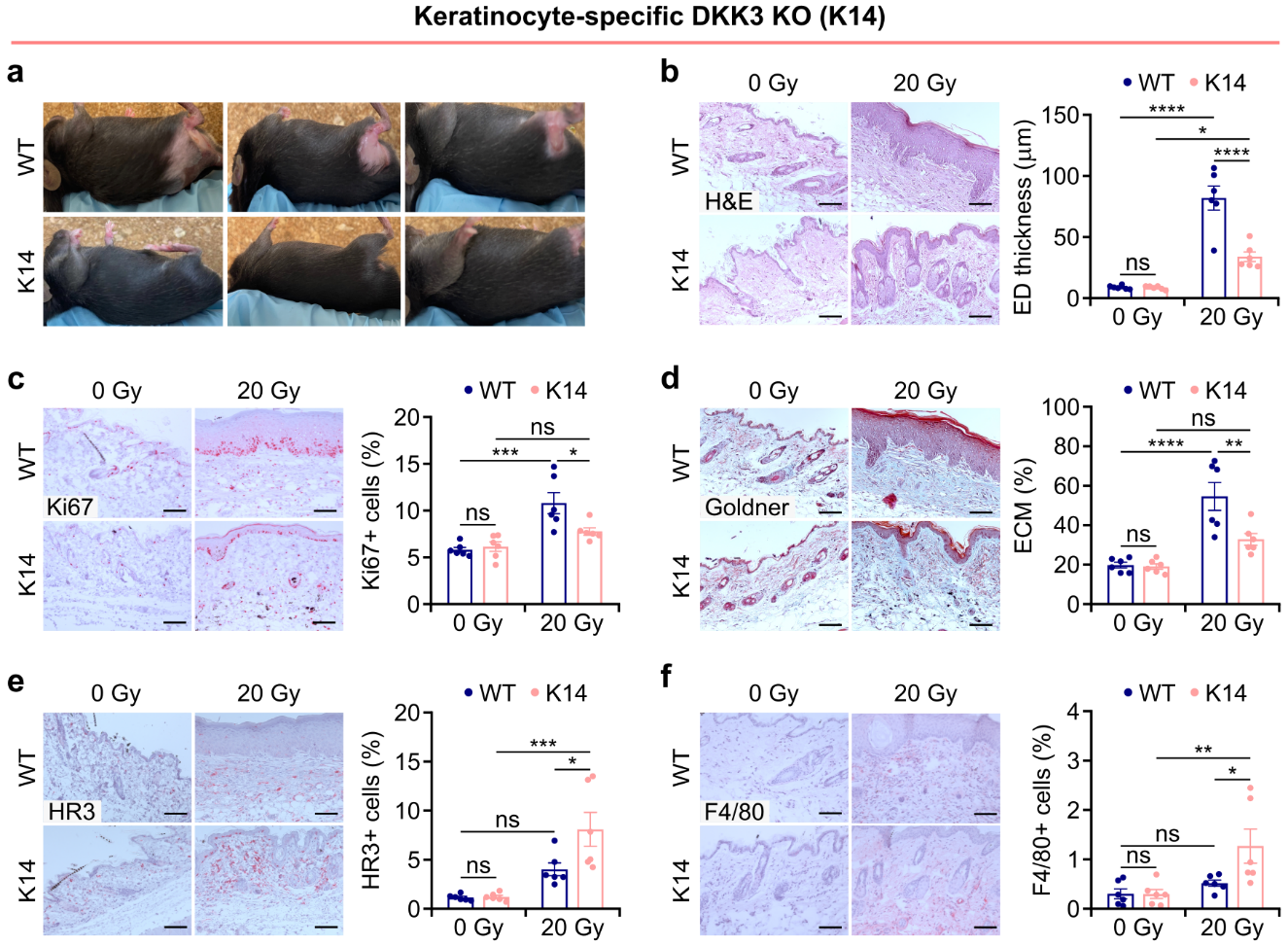
**

**Supplementary Figure 7. Keratinocyte-specific DKK3 knockout mitigates radiation-induced skin toxicity in the hind limb mouse model.** Wildtype (WT) and keratinocyte-specific DKK3 knockout (K14) mice (*n* = 6/group) were analyzed 4 weeks after 20 Gy irradiation of one hind limb versus the unirradiated contralateral hind limb (0 Gy). (**a**) K14 mice were protected from radiation-induced alopecia. (**b-f**) Representative images (left panels) and quantifications (right panels) of skin sections stained for the indicated markers are shown. ED: epidermis. Scale bars: 50 μm. The data are presented as the means ± SEMs. Statistical analysis was performed via two-way ANOVA with Tukey's multiple comparisons test, *P<0.05, **P<0.01, ***P<0.001, ****P<0.0001.

Supplementary Figure 8.

**
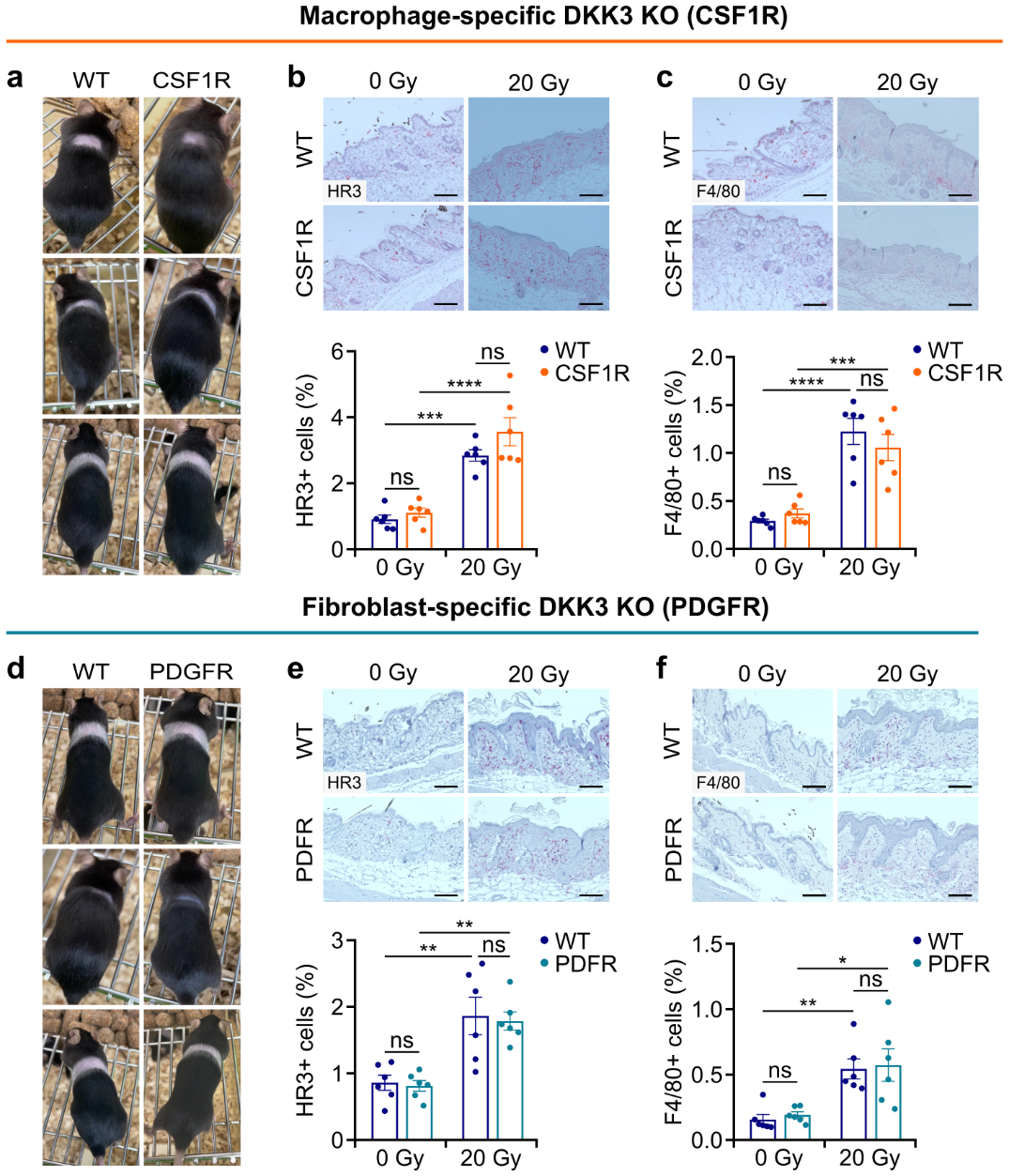
**

**Supplementary Figure 8.** **Macrophage- and fibroblast-specific DKK3 knockout have no significant effects on monocyte or macrophage infiltration.** (**a-c**) Macrophage-specific DKK3 knockout (CSF1R) mice and (**d-f**) fibroblast-specific DKK3 knockout (PDGFR) mice were analyzed 4 weeks after 20 Gy thoracic irradiation (*n* = 6/group). Neither (**a**) CSF1R nor (**b**) PDGFR mice were protected from radiation-induced alopecia. Both knockouts had no influence on radiation-induced infiltration of monocytes/macrophages, as evidenced by representative images (top panels) and quantifications (bottom panels) of skin sections stained for (**b,e**) HR3 and (**c,f**) F4/80. Scale bars: 50 μm. The data are presented as the means ± SEMs. Statistical analysis was performed via two-way ANOVA with Tukey’s multiple comparisons test, *P<0.05, **P<0.01, ***P<0.001, ****P<0.0001.

Supplementary Figure 9.

**
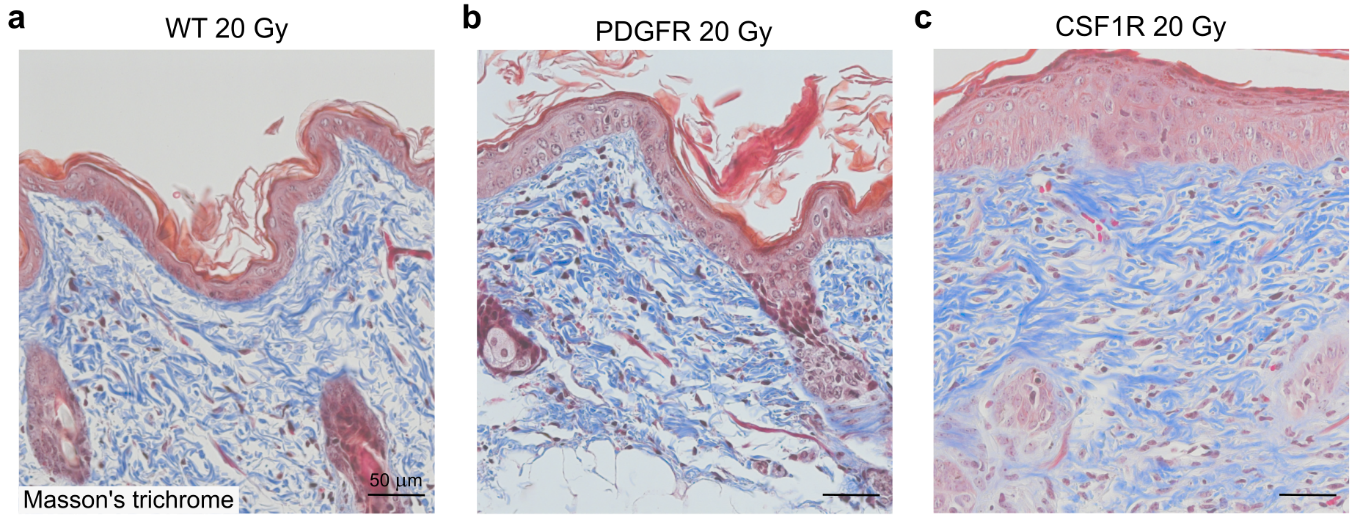
**

**Supplementary Figure 9. Masson’s trichrome staining of skin from fibroblast- and macro-phage-specific DKK3 knockout mice at four weeks after thoracic irradiation with 20 Gy.** (**a**) Irradiated WT and (**b**) fibroblast (PDGFR)-specific DKK3 knockout mice showed similar levels of elevated Masson stain. (**c**) Macrophage (CSF1R)-specific knockout mice showed enhanced Masson stain.

Supplementary Figure 10.

**
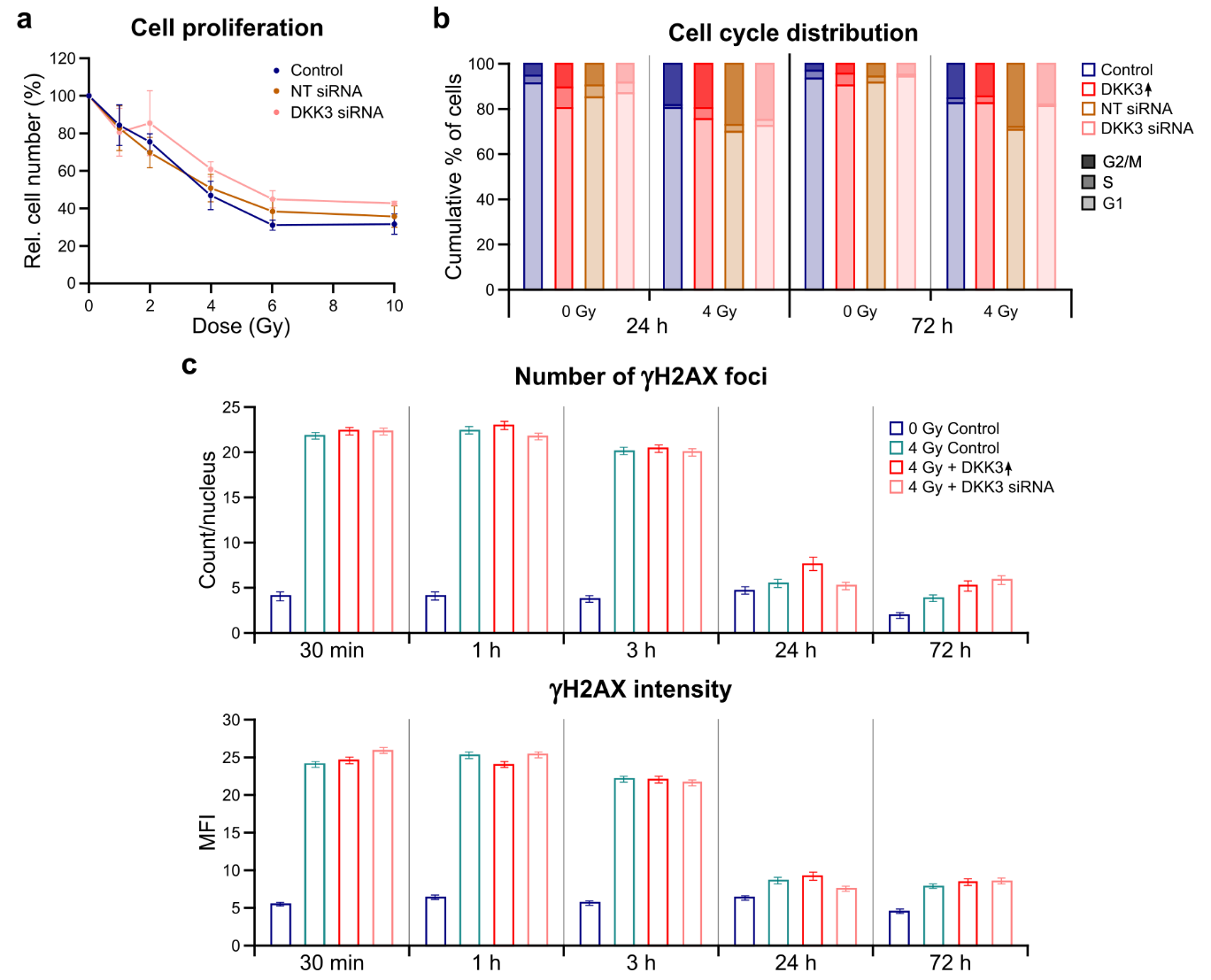
**

**Supplementary Figure 10. Intrinsic radiosensitivity of N/TERT-1 keratinocytes** **is not significantly changed by altered DKK3 expression.** N/TERT-1 keratinocytes with doxycycline (Dox)-inducible DKK3 overexpression were irradiated with 0 or 4 Gy 24 h after siRNA-mediated DKK3 knockdown (DKK3 siRNA) or Dox stimulation (DKK3↑). Unstimulated cells or cells transfected with non-targeting siRNA (NT siRNA) were included as controls. (**a**) Cell number/proliferation 72 h post-irradiation (*n* = 3/dose). (**b**) Average proportions of cells in G1 (lower bars), S (middle bars) and G2/M (upper bars) phase depicted as cumulative percentages at 24 h and 72 h after irradiation (*n* = 3). (**c**) Microscopic evaluation of DNA double-strand break (DSB)-specific γH2AX foci counts/nucleus and γH2AX intensity (*n* = 175-300 nuclei/condition). The data in (a) and (c) are presented as the means ± SEMs.

Supplementary Figure 11.

**
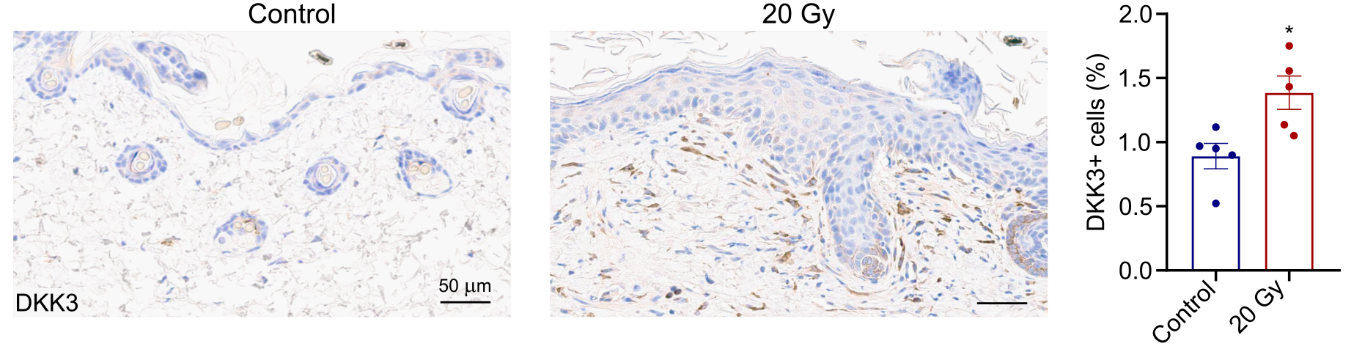
**

**Supplementary Figure 11. Radiation increases DKK3 expression in mouse skin.** Immuno-histochemistry staining of DKK3 in skin sections of WT mice 4 weeks after thorax irradiation with 20 Gy. Scale bars: 50 µm. The data are presented as the means ± SEMs. Statistical analysis was performed via one-way ANOVA with Tukey’s multiple comparisons test, *P<0.05 compared with the control.

Supplementary Figure 12.

**
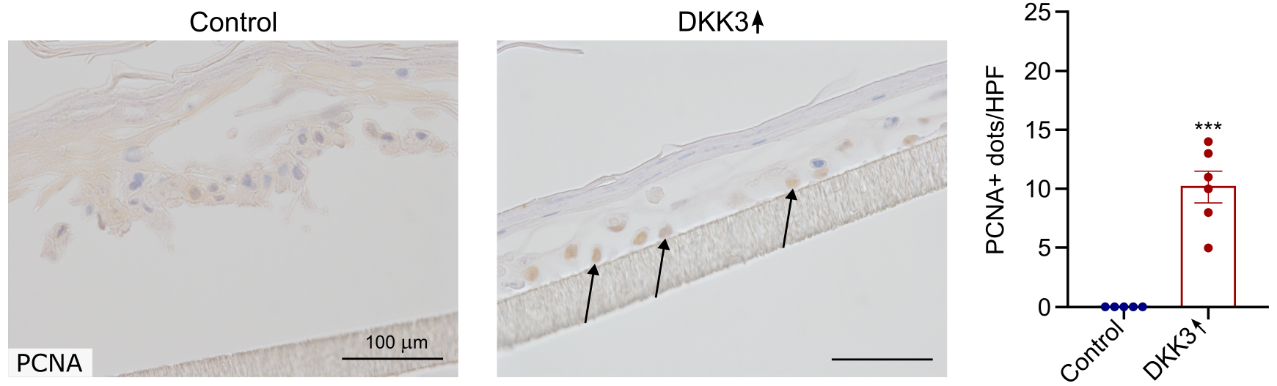
**

**Supplementary Figure 12. DKK3 induction upregulates PCNA in keratinocytes in the 3D skin model.** Immunohistochemistry staining in 3D skin model sections shows expression of the proliferation marker PCNA after DKK3 induction. HPF: high power field. Scale bars: 100 µm. The data are presented as the means ± SEMs. Statistical analysis was performed via Student’s t test with unequal variances, ***P<0.001 compared with the control.

Supplementary Figure 13.


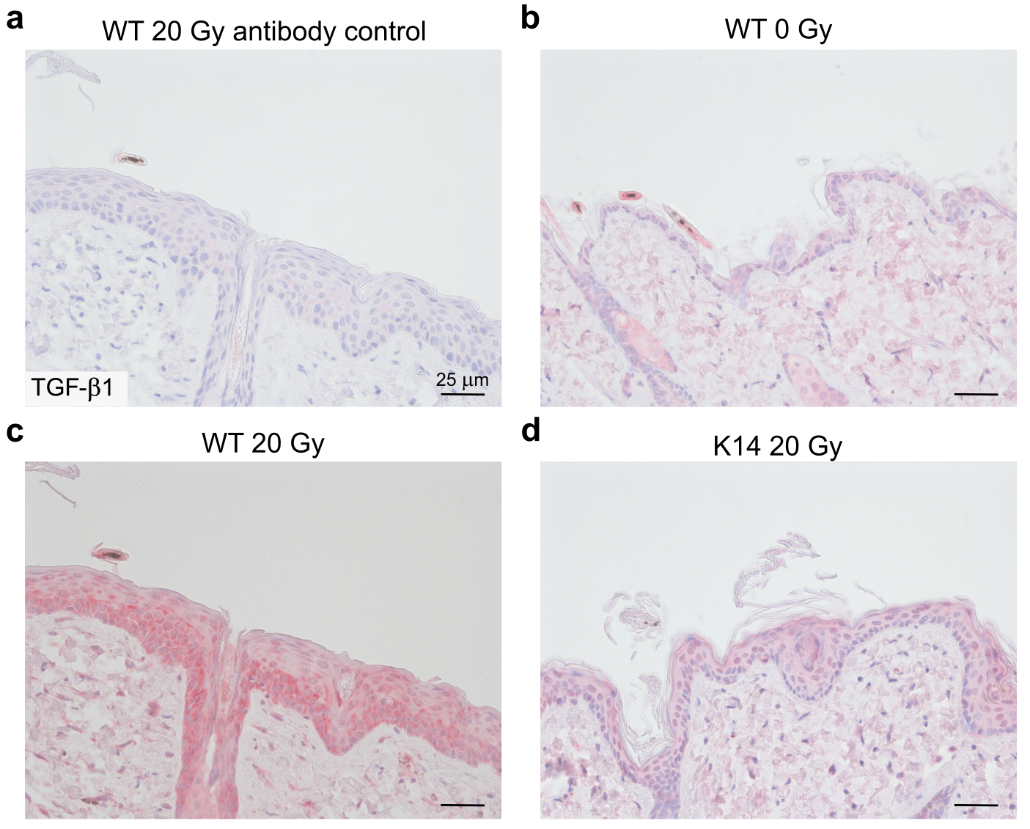


**Supplementary Figure 13. Radiation-induced TGF-β1 expression in the skin, which occurs mainly in basal keratinocytes, is reduced in keratinocyte-specific DKK3 knockout mice.** (**a-c**) Immunohisto­chemistry (IHC) staining of TGF-β1 in skin sections of wildtype (WT) mice 4 weeks after hind limb irradiation with 20 Gy. (**d**) Keratinocyte-specific DKK3 knockout (K14) mice show reduced TGF-β1 expression compared with WT mice at four weeks after irradiation. Antibody control: staining without primary antibody. Scale bars: 25 µm.

Supplementary Figure 14.

**
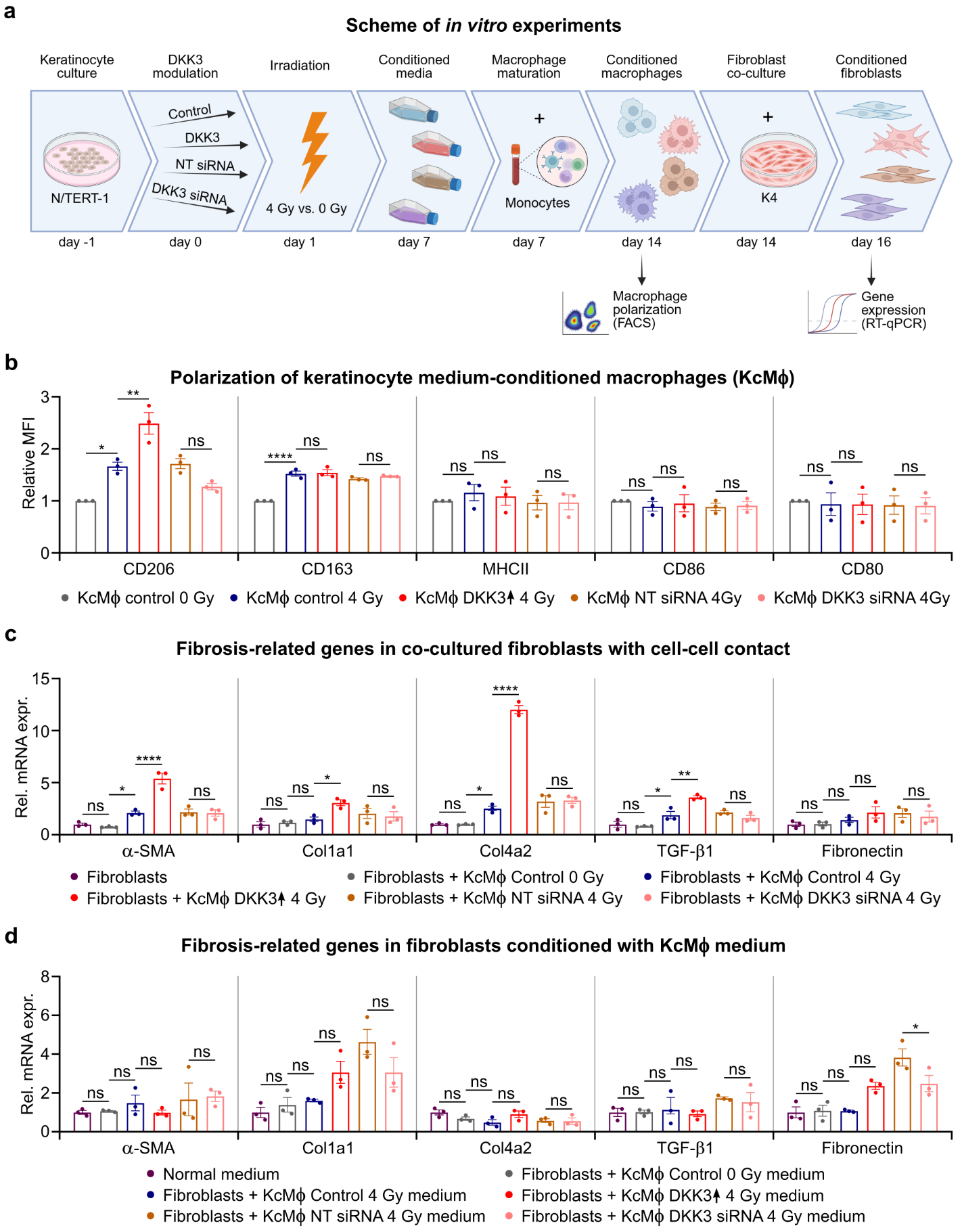
**

**Supplementary Figure 14.** **Radiation partially mimics the effects of DKK3 overexpression in keratinocytes, particularly in promoting macrophage polarization and myofibroblast activation *in vitro*.** (**a**) N/TERT-1 keratinocytes with doxycycline (Dox)-inducible DKK3 overexpression were irradiated with 0 or 4 Gy 24 h after siRNA-mediated DKK3 knockdown (DKK3 siRNA) or Dox stimulation (DKK3↑). Unstimulated cells or cells transfected with non-targeting siRNA (NT siRNA) were included as controls. At 7 days after DKK3 modulation cytokine and growth factor expression was quantified in the cells by RT-qPCR. Conditioned media were harvested for multiplex antibody-based protein profiling and for the maturation of macrophages from freshly isolated monocytes from human PBMCs for another 7 days. Keratinocyte-conditioned macrophages (KcMϕ) were characterized based on surface marker expression. K4 fibroblasts were either co-cultured with KcMϕ or cultured in KcMϕ supernatants only for 2 days. The expression of fibrosis-related genes was evaluated in these conditioned fibroblasts. (**b**) M2-associated surface marker CD206 (FACS) was increased in macrophages conditioned with media of irradiated DKK3 KcMϕ DKK3↑ keratinocytes (KcMϕ DKK3↑ 4 Gy). (**c**) Fibrosis-related gene expression (RT-qPCR) in K4 fibroblasts directly co-cultured (cell-cell contact) with conditioned macrophages. Only macrophages cultured with DKK3-overexpressing keratinocytes (KcMϕ DKK3↑ 4 Gy), but not in DKK3 knockdown keratinocytes (KcMϕ siRNA 4 Gy) induced pro-fibrotic genes in K4 fibroblasts. (**d**) Fibrosis-related gene expression (RT-qPCR) in K4 fibroblasts was unaffected by KcMϕ supernatants (no cell-cell contact). The data are presented as the means ± SEMs. Statistical analysis was performed via one way ANOVA with Tukey's multiple comparisons test, *P<0.05, **P<0.01, ***P<0.001, ****P<0.0001. Figure 14 was partially created in BioRender. Huber, P. (2025) https://BioRender.com/jafruhh.

Supplementary Figure 15.

**
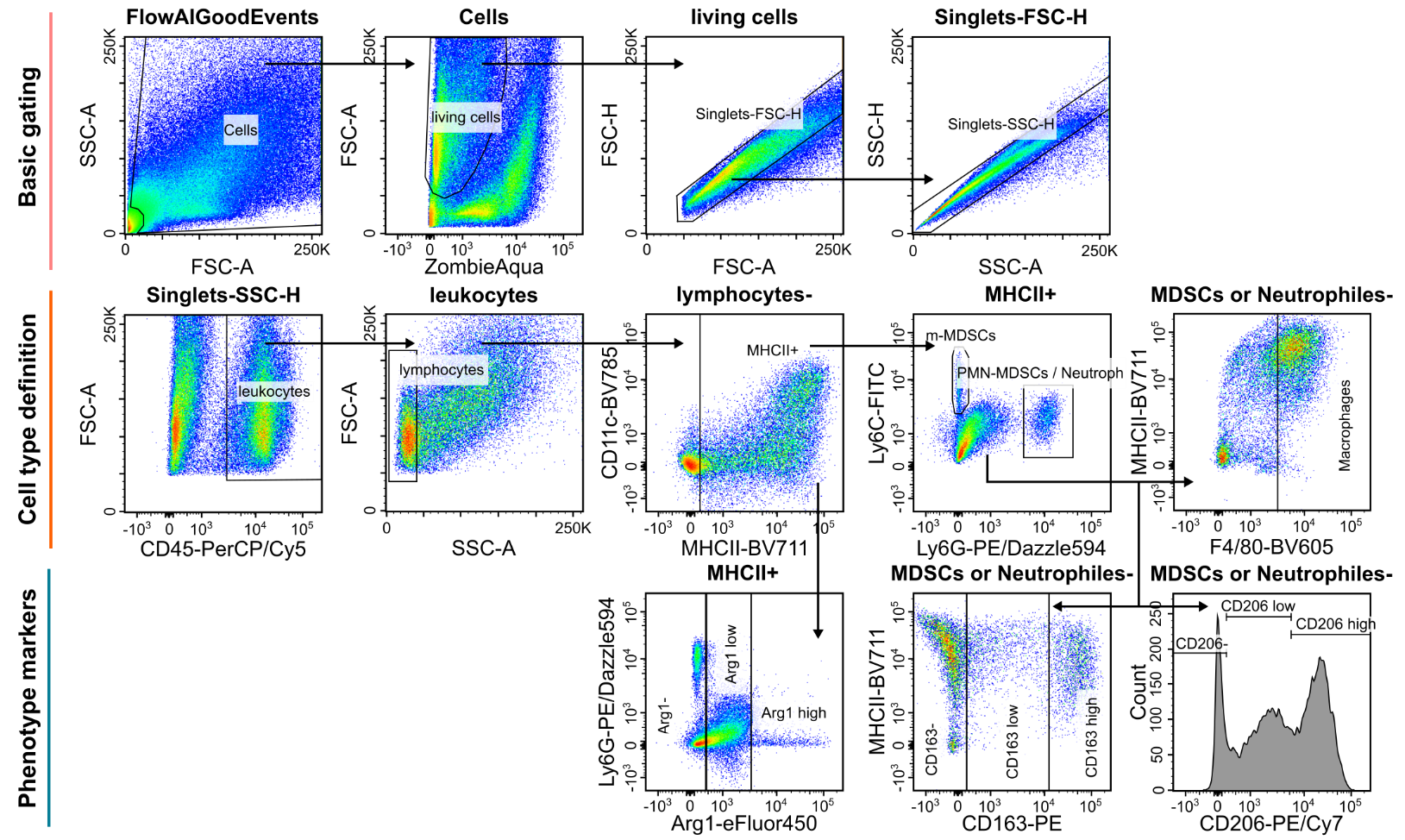
**

**Supplementary Figure 15.** **Gating strategy for flow cytometric analysis of macrophage polarization in fresh mouse skin.** FlowJo 10 software was used for data analysis. Events were filtered with the FlowAI plugin for quality control, low-SSC/lowFSC events were excluded, ZombieAqua-negative living cells were gated and doublets/cell clumps were removed in two steps based on FSC-H vs. FSC-A and SSC-H vs. SSC-A. CD45-positive leukocytes were gated and low-SSC lymphocytes removed before further gating of MHCII-positive cells. Monocytic myeloid-derived suppressor cells (mMDSCs) and polymorphonuclear MDSCs (PMN-MDSCs)/neutrophiles were excluded based on their Ly6C and Ly6G expression. F4/80-positive macrophages were gated and further characterized by their expression levels of arginase 1 (Arg1), CD163 and CD206 using thresholds defined within the indicated populations.

Supplementary Figure 16.

**
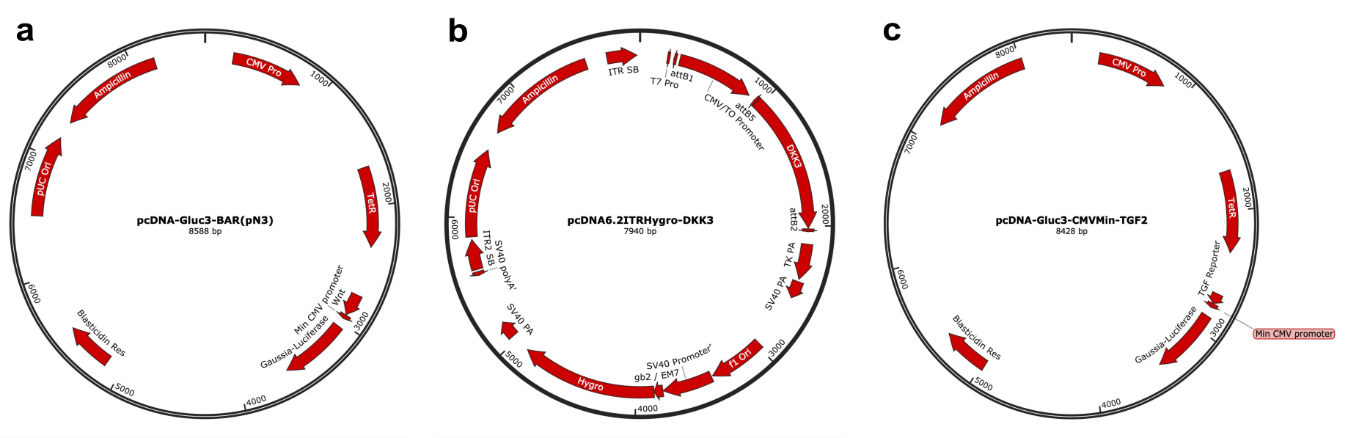
**

**Supplementary Figure 16. Plasmid maps for reporter cells.** (**a**) pSBTR.Wnt (Addgene, #79482), (**b**) pcDNA6.2ITR-Zeo-CMV/TO-DKK3 plasmid, and (**c**) pSBTR.TGF plasmid (Addgene, #79483).

Supplementary Figure 17.

**
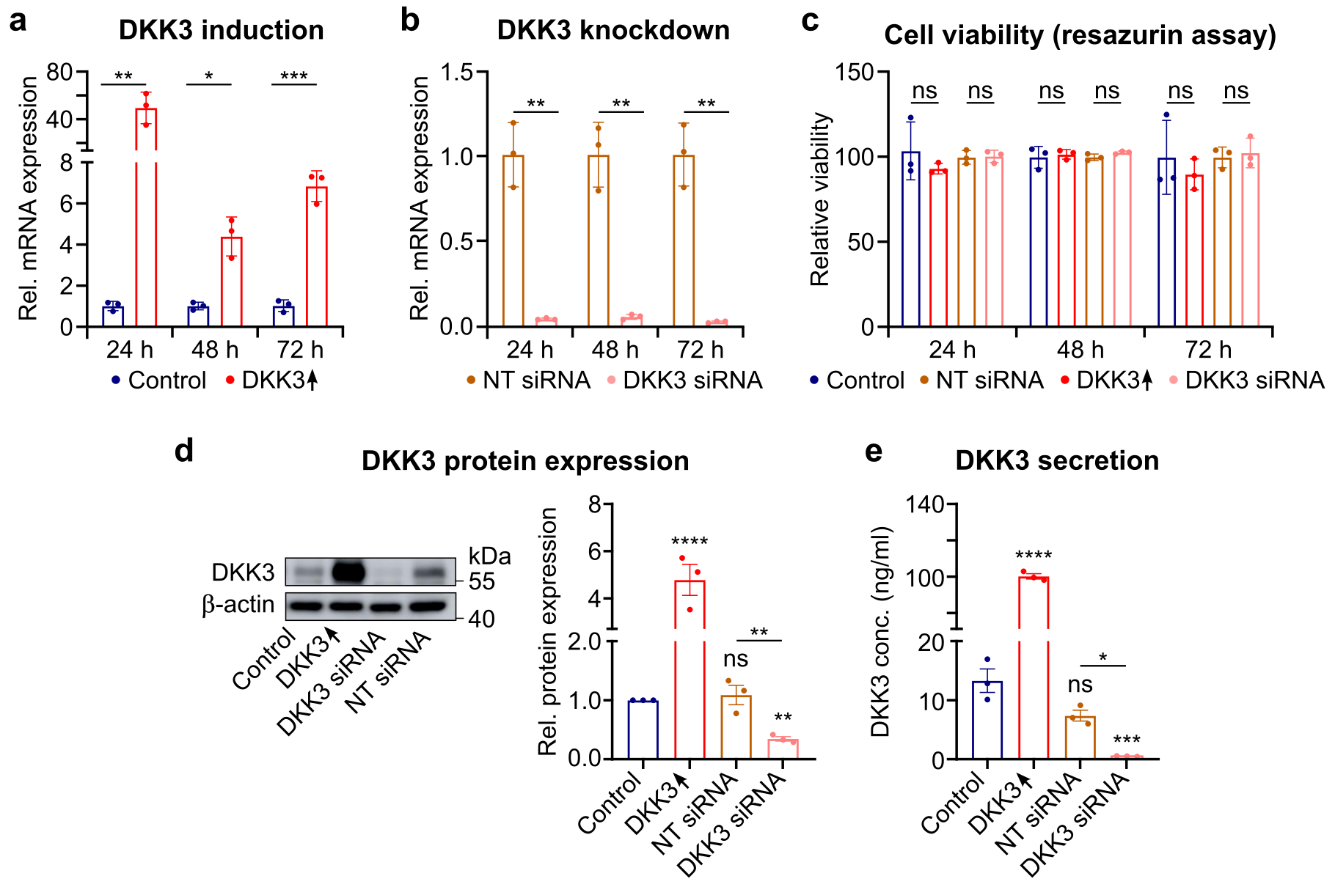
**

**Supplementary Figure 17.** **Validation of the DKK3 modulation system.** DKK3 was knocked down using siRNA (DKK3 siRNA) or overexpressed by doxycycline (Dox) stimulation (DKK3↑) in N/TERT‑1 keratinocytes with Dox-inducible DKK3 overexpression and canonical Wnt reporter. Transfection with non-targeting (NT) siRNA and unstimulated cells (control) were included as references. DKK3 gene expression was measured by RT-qPCR at 24, 48 and 72 h after (**a**) induction or (**b**) knockdown and (**c**) the resazurin assay (see supplementary methods) was used to control cell viability. In addition, DKK3 protein levels were measured at 48 h after modulation in the cells by (**d**) Western blot and (**e**) in supernatants by ELISA. The data are presented as the means ± SEMs. Statistical analysis was performed via (a-c) two-tailed Student’s t tests with Holm-Šidák’s multiple comparison test or (d,e) one-way ANOVA with Tukey’s multiple comparisons test, *P<0.05, **P<0.01, ***P<0.001, ****P<0.0001 compared with the control or as indicated.

Supplementary Figure 18.

**
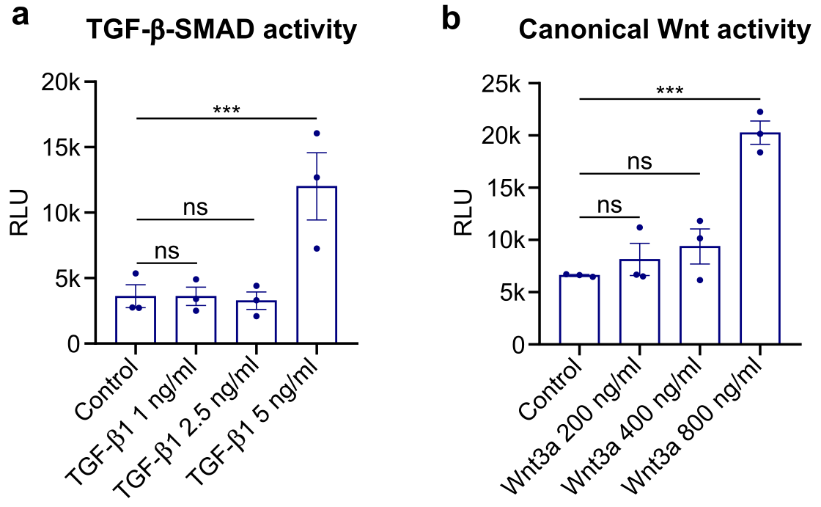
**

**Supplementary Figure 18.** **Validation of the TGF-β-SMAD reporter in K4 fibroblasts and the canonical Wnt reporter in N/TERT-1 keratinocytes.** (**a**) TGF-β-SMAD activity (*Gaussia* luciferase assay) in culture media at 72 h after stimulation of K4 fibroblasts with different concentrations of recombinant TGF-β1. (**b**) Canonical Wnt activity (*Gaussia* luciferase assay) in culture media at 72 h after stimulation of N/TERT1 cells with different concentrations of Wnt3a. RLU: relative light units. The data are presented as the means ± SEMs. Statistical analysis was performed via one-way ANOVA with Tukey’s multiple comparisons test, ***P<0.001.

Supplementary Figure 19.

**
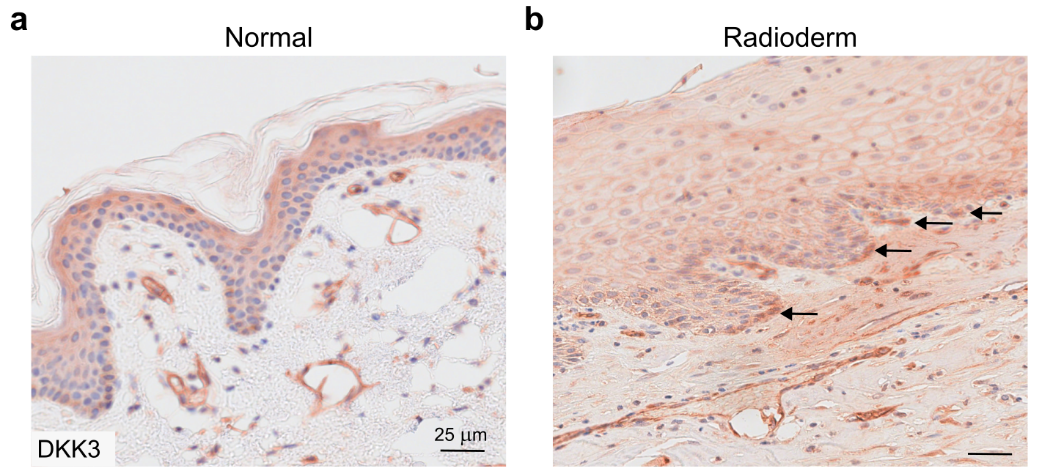
**

**Supplementary Figure 19.** **Validation of enhanced DKK3 expression in human basal keratinocytes following irradiation.** (**a**) Immunohistochemistry staining of non-irradiated human skin shows rare DKK3 expression in the basal keratinocyte layer. (**b**) Irradiated skin shows epithelial hyperplasia and increased expression of DKK3 in basal keratinocytes.

Supplementary Figure 20.

**
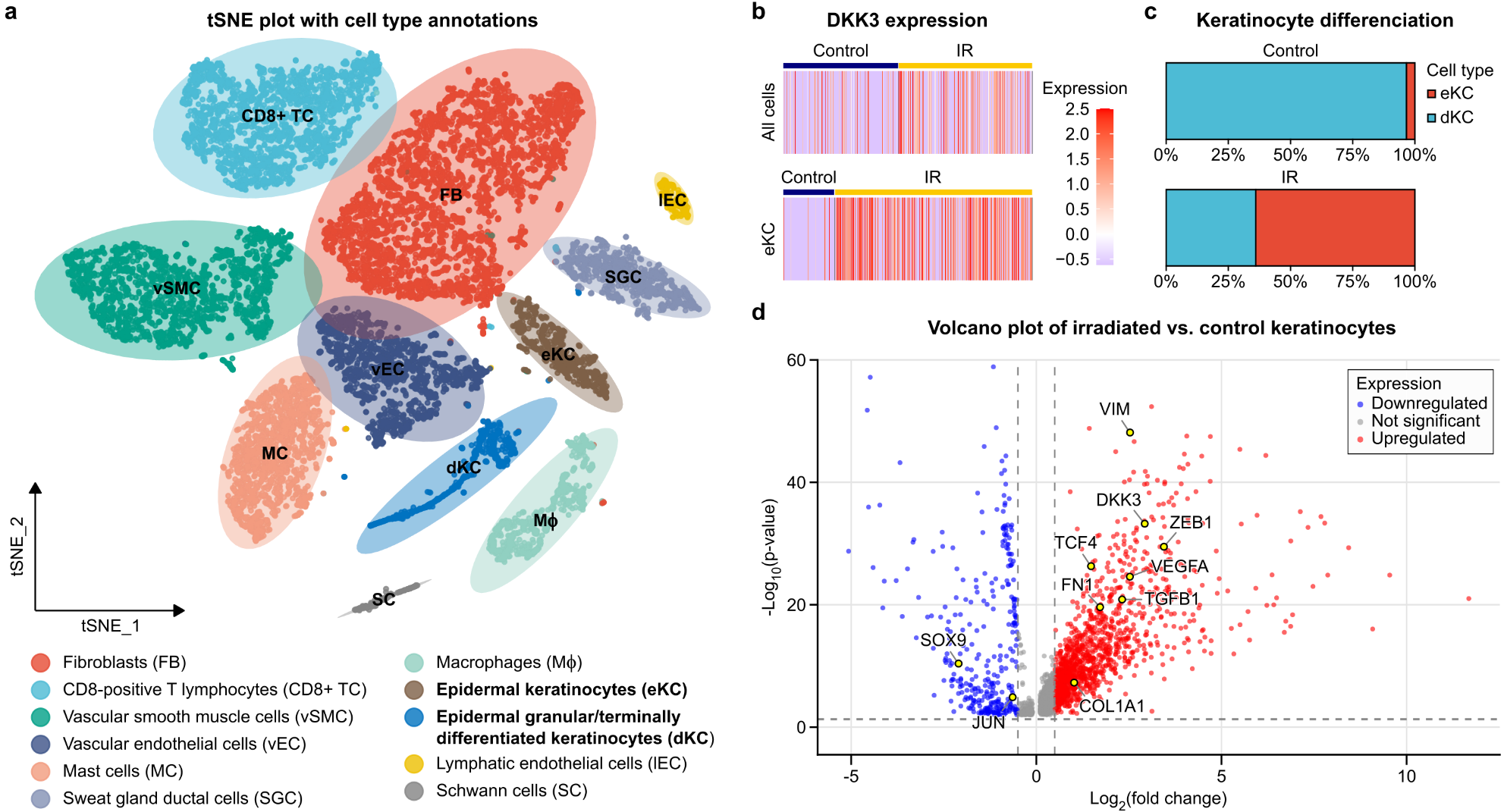
**

**Supplementary Figure 20. Single-cell RNA sequencing of irradiated vs. nonirradiated human whole skin samples from the public GSE193807 dataset.^3^** (**a**) Cell-clustering tSNE plot. Keratinocyte subpopulations: epidermal keratinocytes (eKC; undifferenti-ated, proliferative state); epidermal granular keratinocytes (dKC; differentiated, barrier-forming state). (**b**) Heatmap of DKK3 expression in all cells (upper panel) and in epidermal keratinocytes (lower panel) in the irradiated (IR) and non-irradiated (Control) group. (**c**) Proportion of epidermal and differentiated keratinocytes after IR. (**d**) Volcano plot showing the effects of irradiation on gene expression. Indicated are fibrosis-associated and Wnt signaling-related genes significantly regulated by irradiation.

Supplementary Figure 21.


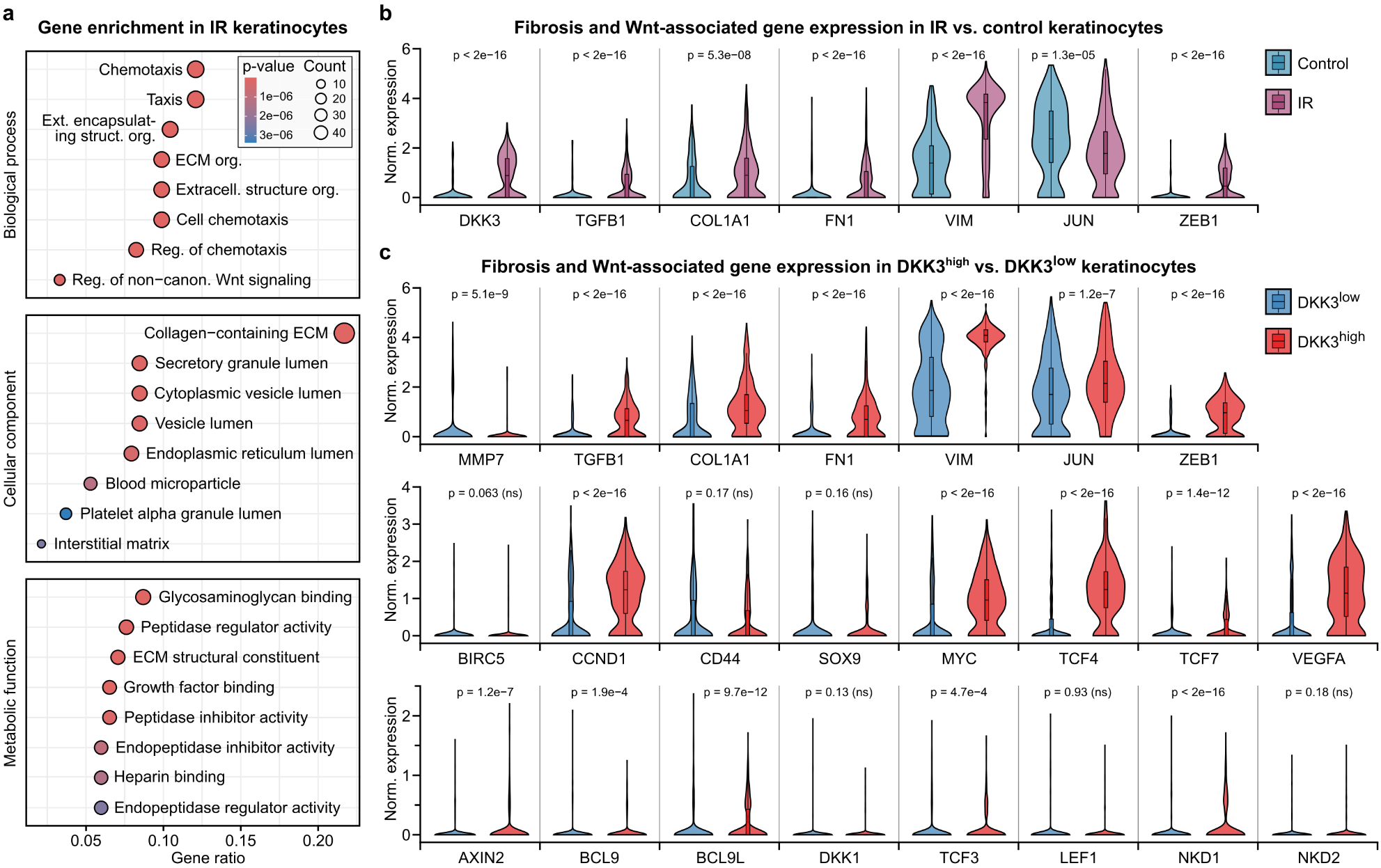


**Supplementary Figure 21.**  **Further analysis of keratinocytes in the GSE193807 dataset.^3^** (**a**) Gene enrichment after IR. (**b**) Violin plots of fibrosis- and Wnt-associated gene expression in IR vs. control and (**c**) DKK3^high^ vs. DKK3^low^ keratinocytes (cutoff = 0.764).

Supplementary Table 1. Gene regulation by DKK3 overexpression in keratinocytes.

| **Symbol** | **Gene name** | **Fold change** | **P-adj** |
| --- | --- | --- | --- |
| MMP7 | Matrix metallopeptidase 7 | 7.0 | 0.002 |
| TIMP2 | TIMP metallopeptidase inhibitor 2 | 4.4 | 0.030 |
| CXCL2 | Chemokine (C-X-C motif) ligand 2 | 2.9 | 0.049 |
| THBS1 | Thrombospondin 1 | 2.2 | 0.011 |
| FN1 | Fibronectin 1 | 2.1 | 0.006 |
| TCF7L1 | Transcription factor 7-like 1 | 2.1 | 0.049 |
| SERPINF1 | Serpin peptidase inhibitor, clade F | 2.0 | 0.010 |
| PTCH1 | Patched 1 | 1.9 | 0.034 |
| ACSL4 | Acyl-CoA synthetase long-chain family member 4 | 1.7 | 0.020 |
| LAMA3 | Laminin, alpha 3 | 1.7 | 0.010 |
| SDC3 | Syndecan 3 | 1.6 | 0.001 |
| MAML2 | Mastermind-like transcriptional coactivator 2 | 1.6 | 0.044 |
| TGF-β1 | Transforming growth factor, beta 1 | 1.6 | 0.020 |
| SERPINE1 | Serpin peptidase inhibitor, clade E 1 | 1.5 | 0.009 |
| IFN-γ | Interferon gamma | 1.5 | 0.001 |
| TGFBR1 | Transforming growth factor, beta receptor 1 | 1.5 | 0.006 |
| IL-6 | Interleukin 6 | 1.5 | 0.050 |
| CPT1A | Carnitine palmitoyltransferase 1A (liver) | 1.5 | 0.030 |
| FGF-2 | Fibroblast growth factor 2 | 1.5 | 0.016 |
| IL-4 | Interleukin 4 | 1.4 | 0.020 |
| SKP2 | S-phase kinase-associated protein 2 | 1.4 | 0.022 |
| EGFR | Epidermal growth factor receptor | 1.4 | 0.030 |
| TRIB3 | Tribbles pseudokinase 3 | -5.0 | 0.002 |
| DDIT3 | DNA-damage-inducible transcript 3 | -2.3 | 0.003 |
| VEGFA | Vascular endothelial growth factor A | -2.2 | 0.001 |
| LIPG | Lipase, endothelial | -2.0 | 0.022 |
| ATF4 | Activating transcription factor 4 | -1.9 | 0.001 |
| ARHGEF2 | Rho/Rac guanine nucleotide exchange factor (GEF) 2 | -1.8 | 0.023 |
| IL-10 | Interleukin 10 | -1.7 | 0.043 |
| CXCL8 | Chemokine (C-X-C motif) ligand 8 | -1.5 | <0.001 |
| PDGF-BB | Platelet Derived Growth Factor Subunit B | -1.5 | 0.006 |
| CXCL10 | Chemokine (C-X-C motif) ligand 10 | -1.4 | 0.022 |

Fold changes of differentially expressed genes in N/TERT-1 keratinocytes with doxycycline (Dox)-inducible DKK3 overexpression and canonical Wnt reporter at 24 h after Dox stimulation versus basal DKK3 expression in unstimulated controls (nCounter analysis, see also heatmap in Figure 5a). P values are adjusted for multiple testing correction (Benjamini-Hochberg). Note: negative numbers are transformed values -1/fold change (see Rosalind documentation).

Supplementary Table 2. Pathway activation
by DKK3 overexpression in keratinocytes.

| **Pathway enrichment** | **Z-score** |
| --- | --- |
| Th2 Differentiation | 5.1583 |
| Platelet Degranulation | 4.2062 |
| Insulin Signaling | 4.0073 |
| Notch | 3.5898 |
| MAPK Cell Stress | 3.2591 |
| PI3K/Akt | 3.2543 |
| EMT | 3.2223 |
| Adenosine Pathway | 3.1437 |
| Angiogenesis | 3.0907 |
| Insulin Resistance | 3.0880 |

Pathway enrichment analysis was applied for genes differentially regulated by DKK3 overexpression in keratinocytes versus unstimulated controls (see Table S1) using the ROSALIND® NanoString nCounter Gene Expression tool (https://rosalind.onramp.bio/). Positive z-scores reflect the strength of pathway activation. Analysis shows changes in pathways linked to fibrotic development, Wnt activation and cell stress including T helper 2 immune response, MAPK stress signaling and epithelial to mesenchymal transition (EMT).

Supplementary Table 3. Antibodies for immunohistology

| **Antibody** | **Order no.** | **Company** | **Dilution** |
| --- | --- | --- | --- |
| DKK3 | ab186409 | abcam | 1:200 |
| Ki 67 | IHC-00375 | Bethyl | 1:300 |
| F4/80 | BM4007 | Acris | 1:100 |
| HR3 | BM4016 | Acris | 1:200 |
| CD3 | DIA-3003 | Dianova | 1:50 |
| α-SMA | A2547 | Sigma- Aldrich | 1:200 |
| FoxP3 | 14-5773-82 | Invitrogen/eBioscience | 1:50 |
| Keratin 5 | 905503 | Biologend | 1:200 |
| Keratin 10 | 905403 | Biologend | 1:200 |
| Keratin 16 | MBS9600127 | MyBioSource | 1:50 |
| Human DKK3 | AF1118 | R&D | 1:200 |
| PCNA | MABE 288 | Merck | 1:100 |
| TGF-beta1 | LS-C 161825 | LSBio | 1:400 |

Supplementary Table 4. Antibodies for flow cytometric
analysis of mouse skin immune cell infiltrate.

| **Antibody** | **Order no.** | **Company** | **Dilution** |
| --- | --- | --- | --- |
| Ly6C- FITC | 128005 | Biolegend | 1:640 |
| Ly6G- PE/Dazzle594 | 127647 | Biolegend | 1:320 |
| CD11b- Alexa Fluor 700 | 101222 | Biolegend | 1:640 |
| CD11c- Brilliant Violet 785 | 117335 | Biolegend | 1:40 |
| F4/80- BV605 | 123133 | Biolegend | 1:40 |
| MHC-II (I-A/I-E)- BV711 | 123133 | Biolegend | 1:160 |
| iNOS- APC | 17-5920-82 | ThermoFisher | 1:320 |
| Arg1- eFluor450 | 48-3697-80 | ThermoFisher | 1:20 |
| CD206- PE/Cy7 | 141719 | Biolegend | 1:80 |
| CD163- PE | 155307 | Biolegend | 1:80 |
| CD45- PerCp-Cy5.5 | 103131 | Biolegend | 1:100 |
| Zombie aqua (FVD) | 423105 | Biolegend | 1:100 |

Supplementary Table 5. Primer sequences.

| **Gene** | **Forward primer** | **Reverse primer** |
| --- | --- | --- |
| 18s rRNA | GCAATTATTCCCCATGAACG | AGGGCCTCACTAAACCATCC |
| DKK3 | GTGCATCATCGACGAGGACTGT | TGGTCTCCACAGCACTCACTGT |
| TGF-β1 | TACCTGAACCCGTGTTGCTCTC | GTTGCTGAGGTATCGCCAGGAA |
| GM-CSF | GGAGCATGTGAATGCCATCCAG | CTGGAGGTCAAACATTTCTGAGAT |
| IL-1 β | CCACAGACCTTCCAGGAGAATG | GTGCAGTTCAGTGATCGTACAGG |
| TNF-α | CTCTTCTGCCTGCTGCACTTTG | ATGGGCTACAGGCTTGTCACTC |
| CXCL8 | TATCCAGAGGCTGGAGAGCTAC | TGGAATCCCTGACCCATCTCTC |
| CXCL10 | GGTGAGAAGAGATGTCTGAATCC | GTCCATCCTTGGAAGCACTGCA |
| PDGFA | CAGCGACTCCTGGAGATAGACT | CGATGCTTCTCTTCCTCCGAATG |

Supplementary Table 6. RTOG/EORTC radiation morbidity scoring criteria.^1^

| **Score** | **Chronic morbidity** |
| --- | --- |
| 0 | None |
| 1 | Slight atrophy; pigmentation change; some hair loss |
| 2 | Patch atrophy; moderate telangiectasia; total hair loss |
| 3 | Market atrophy; gross telangiectasia |
| 4 | Ulceration |
| 5 | Death |

RTOG: Radiation Therapy Oncology Groups. EORTC: European Organization for Research and Treatment of Cancer.

Supplementary References

1. Cox, J. D., Stetz, J. & Pajak, T. F. Toxicity criteria of the Radiation Therapy Oncology Group (RTOG) and the European Organization for Research and Treatment of Cancer (EORTC). *Int J Radiat Oncol Biol Phys* **31**, 1341-1346 (1995).

2. Li, L. DKK3 driven Wnt pathway in radiation-induced inflammatory and fibrosing skin injury*.* *Doctoral degree of the human biology thesis, Ludwig-Maximilians-University Munich, Germany*, (2023).

3. Yan, T. *et al.* Single-cell RNA-Seq analysis of molecular changes during radiation-induced skin injury: the involvement of Nur77. *Theranostics* **14**, 5809-5825 (2024).
