# Supplementary figures and images for: Wnt-associated DKK3 in keratinocytes mediates radiation-induced hyperplasia, dermatitis and skin fibrosis
Source: Signal Transduct Target Ther. 2026 Feb 2;11:41. doi: 10.1038/s41392-025-02541-z (PMC12864833; doi:10.1038/s41392-025-02541-z)

# Uncut Western Blot images

**Figure 5c**

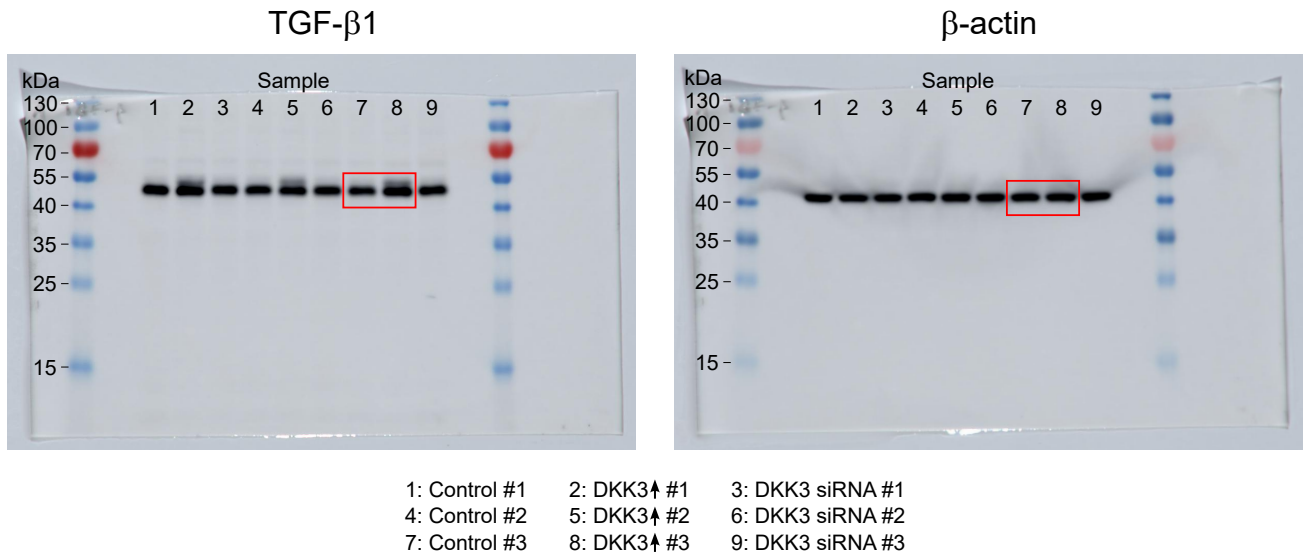

**Supplementary Fig. 17d**

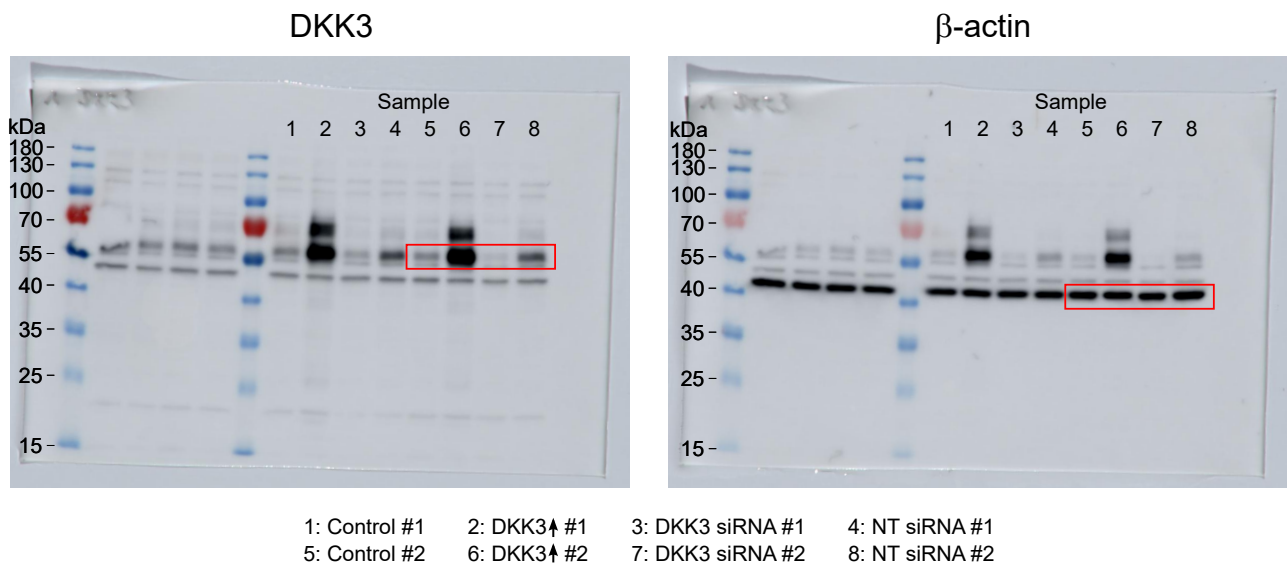

Supplement: Supplementary file 2 — Supplementary Data 1_uncropped WB [file 41392_2025_2541_MOESM2_ESM.pdf]
